# T‐CLASS: An Online Tool for the Identification and Classification of Aging and Senescence Using Transcriptome Data

**DOI:** 10.1111/acel.70193

**Published:** 2025-08-14

**Authors:** Seung‐Chul J. Lee, Gee‐Yoon Lee, Sieun S. Kim, Yunkyu Bae, Seokjin Ham, Jooyeon Sohn, Seong Kyu Han, Seung‐Jae V. Lee

**Affiliations:** ^1^ Department of Biological Sciences Korea Advanced Institute of Science and Technology Daejeon South Korea; ^2^ Department of Biological Sciences Inha University Incheon South Korea

**Keywords:** aging, *C. elegans*, classification, longevity, transcriptome, web‐based tool

## Abstract

Transcriptome analysis has become increasingly utilized in aging research. However, the identification of the key molecular changes underlying aging processes and longevity‐promoting regimens from transcriptome data remains challenging. Here, we present Transcriptomic CLassification via Adaptive learning of Signature States (T‐CLASS), an online tool that identifies, from transcriptome data, gene sets of several hundred genes that provide an optimal representation of longevity and aging paradigms. We systematically evaluated the effectiveness of T‐CLASS with diverse datasets, including longevity‐promoting regimens in *Caenorhabditis elegans* , cellular senescence by different means in both cultured mouse primary cells and cultured human cells, and human sarcopenia. We found that T‐CLASS exhibited robust and high classification performance across datasets compared to preexisting machine/deep learning‐based gene selection tools. By focusing our further analysis on longevity‐promoting regimens in *C. elegans* , we showed that T‐CLASS successfully classified transcriptomic changes caused by ten lifespan‐extending small molecules, among which we experimentally validated the effect of rifampicin and atracurium as a proof of principle. Overall, T‐CLASS is an effective and practical tool for uncovering and classifying physiological changes caused by genetic and pharmacological interventions that affect aging.

## Graphical abstract

T‐CLASS is a web‐based tool that selects an optimal gene set to categorize and classify transcriptomic changes associated with aging and longevity.

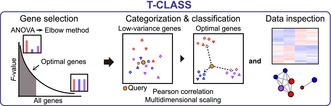

## 1 Introduction

Advances in transcriptomic analysis have enhanced our understanding of the molecular changes underlying various physiological processes. Global gene expression patterns provide insights into molecular and cellular states under different conditions, such as aging, development, disease progression, and therapeutic interventions (Lee, Kim, Chu, et al. 2024; Lee et al. 2021; Sepp et al. 2024; Tae et al. 2024; Uhlen et al. 2017; Yang et al. 2020). To date, numerous studies using microarray and RNA sequencing (RNA‐seq) methods have accumulated vast amounts of transcriptomic data across diverse cellular and organismal contexts. For example, over 150,000 microarray and RNA‐seq datasets are currently deposited into the Gene Expression Omnibus (GEO) database (Barrett and Edgar 2006; Barrett et al. 2013; Clough et al. 2024). However, integrating and classifying physiological changes based on vast amounts of transcriptome data remain challenging.

The identification of appropriate and manageable gene sets that represent changes in physiological states, including longevity and aging, is a major hurdle for biologists to effectively analyze transcriptomic data. Conventional dimensionality reduction methods, such as principal component analysis (PCA) and multidimensional scaling (MDS), use the full sets of genes for analysis, often causing challenges in discerning transcriptomic differences due to residual uninformative components, such as noise (Lin and Fukuyama 2024). Moreover, the scopes of influenced genes can vary widely depending on factors such as effects in gene networks and downstream pathways resulting from the physiological changes. Therefore, choosing appropriate gene sets for further analysis is crucial for proper biological interpretation of physiological states.

Longevity, defined as an extension of lifespan, is influenced by a complex interplay of genetic and environmental factors that affect aging processes at the organism level. Longevity‐promoting regimens have been identified across various species. For example, the reduced insulin/IGF‐1 signaling pathway (rIIS) is conserved from nematodes to fruit flies, mice, and humans (C. J. Kenyon 2010). Additionally, other longevity‐promoting regimens, such as mild reductions in mitochondrial function (rMF) and dietary restriction (DR), are also conserved across multiple species (C. Kenyon 2005; Lee et al. 2015; Pan and Finkel 2017; Partridge et al. 2011; Zong et al. 2024). Transcriptome analysis has become a powerful tool in longevity and aging research, enabling the examination of global gene expression changes that occur over lifespan (Gao et al. 2024; Lee, Ham, Sohn, et al. 2024; Loose et al. 2022). However, the comprehensive integration of transcriptomic changes to dissect longevity‐promoting regimens remains challenging to researchers due to its complexity and irrelevant residual factors. These limitations highlight the need for analytical frameworks that visualize transcriptional landscapes associated with longevity and aging by identifying appropriate gene sets representing these states.

In this work, we present Transcriptomic CLassification via Adaptive learning of Signature States (T‐CLASS), an online analysis platform that visualizes transcriptomic differences associated with specific physiological states, in particular, longevity and aging, through identifying representative gene sets with parametric *F*‐statistics, which calculates variance of gene expression across conditions. Our T‐CLASS performed filtering, dimensionality reduction, and visualization of transcriptomic states in a one‐stop online platform. Using gene count‐based processed transcriptomes as query input, we showed that T‐CLASS successfully classifies longevity‐promoting regimens in *Caenorhabditis elegans* , types of senescence in both cultured mouse primary cells and cultured human cells, and human sarcopenia. In addition, by performing leave‐one‐out cross‐validation, we found that classification performance of T‐CLASS is generally high and robust compared to four other representative gene selection tools across the datasets. We further analyzed longevity‐promoting interventions in *C. elegans* by using an optimal gene set obtained with T‐CLASS, which identified transcriptomic changes induced by ten small molecules that promote longevity and classified these based on their mode of action. Moreover, using T‐CLASS, we classified transcriptomic changes caused by treatment with rifampicin and atracurium as a dietary restriction‐induced longevity regimen, which we experimentally validated using molecular genetics with *C. elegans* . With these unique features, T‐CLASS will be invaluable for researchers to pinpoint functional effectors of longevity and aging paradigms with transcriptome data, accelerating research by guiding subsequent functional experiments.

## 2 Methods

### 2.1 Adaptive Identification of Gene Sets Representing Physiological States

**FIGURE 1:**
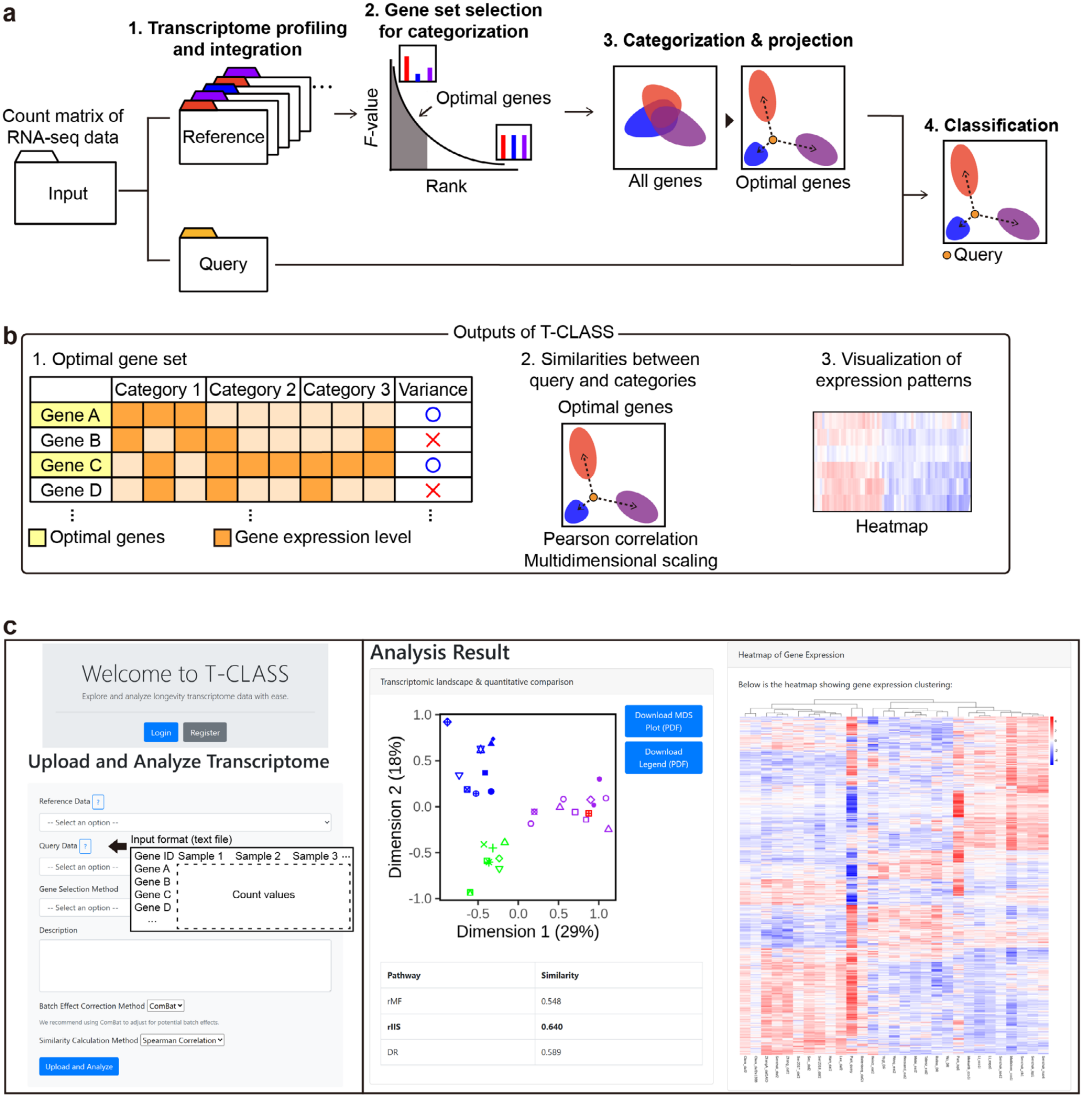
Workflow and features of Transcriptomic CLassification via Adaptive learning of Signature States (T‐CLASS). (a) A schematic diagram of the T‐CLASS. T‐CLASS can be applied across various species. The analysis proceeds based on the following workflow, using the count matrix from RNA sequencing (RNA‐seq) results as input. (1) Generation of reference data for categorization through transcriptome profiling and integration. (2) Selection of the optimal gene set for categorization by applying effective statistical approaches, including analysis of variance (ANOVA) test and elbow method. *F*‐value indicates the degree of variation in gene expression across categories associated with distinct physiological states. (3) Categorization and two‐dimensional projection of transcriptome based on the optimal gene set. (4) Classification of query data by calculating transcriptome similarities among samples based on Spearman correlation. (b) Outputs of T‐CLASS. T‐CLASS analysis is performed in a one‐stop manner, providing users with the following three outputs. (1) Optimal gene set. T‐CLASS calculates the variance (*F*‐value) in gene expression among categories. Genes with higher or lower expression in specific categories were ranked by variance. Genes with higher variance than that at the elbow point were selected as an optimal gene set for categorization. (2) Similarities between query and categories. Using the optimal genes, T‐CLASS performs Multidimensional scaling (MDS) to project the transcriptome similarity among categories onto a two‐dimensional space and calculates Spearman correlation to classify the query among the categories. (3) Visualization of expression patterns. T‐CLASS provides a result of heatmap analysis that visualizes the overall gene expression based on the optimal gene set. (c) T‐CLASS website interface. The T‐CLASS platform allows users to upload query and reference RNA‐seq count data in .txt format. Users can calculate similarity among samples by using Spearman correlation. The interface offers flexible gene selection methods, including T‐CLASS and Boruta, allowing users to customize the gene list for optimized categorization and visualization. See Appendi S2 for the detailed manual of using T‐CLASS.

To identify the optimal and minimal gene sets that appropriately distinguish the physiological state of interest, *F*‐statistics were used. For each gene, an analysis of variance (ANOVA) was performed to compute its *F*‐value (Pavlidis 2003). The *F*‐value represents the variance in gene expression among categories of physiological states of transcriptomes representing different physiological states. Genes were then ranked based on their *F*‐values. Genes with high ranks exhibited higher *F*‐values, indicating that they discriminate the differences among categories more effectively than those with lower *F*‐values. Subsequently, the elbow method was employed to select the minimal number of genes that effectively interpret transcriptome changes across different physiological states (Shalaby et al. 2021). The elbow method identifies the “elbow point” on a curve in which the rate of increase in explained variance of genes sharply decreases. The algorithm calculates the perpendicular distance of each *F*‐value from a straight line drawn between the first and last points on the curve of ranked *F*‐values (Shalaby et al. 2021). The gene corresponding to the maximum perpendicular distance was identified as the elbow point, and the genes with *F*‐values higher than those of the elbow point were determined as the optimal genes for the most effective categorization (Figure 1, gray shading). See Table S1 for the list of published transcriptome data used in this study to identify the optimal and minimal gene sets associated with different physiological states.

### 2.2 Quantification and Visualization of Transcriptomic Differences

Multidimensional scaling (MDS) (Cox and Cox 2000) was used based on the optimal gene sets to project the transcriptomic landscape onto a two‐dimensional plot. The transcriptomic differences were calculated based on a processed count matrix generated from normalized gene expression values. The raw counts were normalized using DESeq2 (v1.28.0.) (Love et al. 2014). The distances among samples were determined by calculating Euclidean distance in gene expression profiles. To assess correlations among samples, Spearman correlation coefficients were obtained by using the pairwise complete observation method, which calculates data pairs with no missing value. This restriction allowed for maximum data utilization, ensuring that the correlation matrix was constructed without introducing bias from missing values, as only complete observations were used. R (v.4.1.0, http://www.r‐project.org) was used for all the data plotting and statistical tests in this study unless stated otherwise.

### 2.3 Machine Learning‐Based Approach

Extreme Gradient Boosting (XGBoost) was utilized following a previous paper (Chen and Guestrin 2016), which randomly selected key genes for categorizing. XGBoost, a robust decision tree algorithm (Li et al. 2022), calculates feature importance scores to identify key genes for categorizing and classification. Genes with a feature importance score greater than 0 were selected for categorizing. The following parameters were used for the analysis: colsample_bytree = 0.9, min_child_weight = 0.5, max_depth = 6, and subsample = 0.8. XGBoost was performed with a fixed seed, because of its inherent randomness that produces different gene sets across runs (Table S1). Boruta, a feature selection method built upon the decision trees classifier (Kursa and Rudnicki 2010), was used to evaluate classification performance. Boruta iteratively compares the importance of real features against permuted shadow features to identify relevant variables for classification. Genes classified as “Confirmed”, defined as those whose importance scores are significantly higher than the maximum importance of permuted shadow features across iterations, were selected as key features. Boruta was used with default settings and a fixed random seed to ensure reproducibility. The Boruta algorithm was implemented on the T‐CLASS website (http://www.t‐class.kaist.ac.kr) to classify query data alongside the T‐CLASS algorithm. The feedforward neural network (FNN) model learns nonlinear representations of gene expression data using a multilayer perceptron with dropout regularization (Candel et al. 2016). Gene importance was computed using internal variable importance measures, and genes with importance values greater than 0 were selected. The architectures were composed of three hidden layers with 256, 128, and 64 units, respectively. A fixed seed was applied to ensure reproducibility. The autoencoder, a dimensionality reduction approach, was implemented with the Keras framework (Gulli and Pal 2017). The autoencoder compresses input features into a latent representation by minimizing reconstruction loss. Genes contributing most to the bottleneck layer were considered important and selected based on weight magnitude. The architecture included an encoder with three layers (256, 128, and 64 units) and a symmetric decoder. A fixed random seed was used to ensure consistent output.

### 2.4 Transcriptome Categorization Using Seurat

Seurat (Hao et al. 2024) was used to evaluate the performance of a widely‐used transcriptome categorizing tool and to compare it with those of T‐CLASS. Raw count values were processed following previous studies (Hwang 2023; Kim, Lim, and Park 2024; Ryu et al. 2023). Count values were transformed into a Seurat object by using CreateSeuratObject. For the function FindVariableGenes, the default parameter was used for the analysis. For functions RunPCA and RunUMAP, the total number of principal components to compute was set as one less than the total number of samples. The following parameters were used for the analysis by using the function RunUMAP: n.neighbors = 5, min.dist = 0.1.

### 2.5 RNA Sequencing and Preprocessing for Test Cases

Raw sequencing data obtained from GEO database were aligned to the *Caenorhabditis elegans* genome WBcel235 (ce11, Ensembl release 103), mouse genome GRCm39 (mm10), and human genome GRCh38 (hg38) by using STAR (v2.7.0e) (Dobin et al. 2013), following previous studies (Ham et al. 2022; Lee, Ham, and Lee 2024; Lee, Ham, Sohn, et al. 2024). Aligned reads to genes and transcripts were quantified by using RSEM (v.1.3.1) (Li and Dewey 2011). Differentially expressed transcripts were identified by using DESeq2 (v1.28.0.) (Love et al. 2014). In merged RNA‐seq data, genes with a count of one or more in at least 80% of samples were prefiltered for downstream analysis. For *C. elegans* longevity transcriptome datasets, 2 out of 27 RNA‐seq datasets were adjusted with upper quartile normalization, followed by using Remove Unwanted Variation from RNA‐Seq Data (RUVSeq) (v.1.22.0) (Risso et al. 2014). The raw counts were normalized using variance‐stabilizing transformation (VST) implemented in DESeq2 and designed to account for variance differences across individual genes (Anders and Huber 2010; Lin et al. 2008). After normalization, batch correction was performed by using “removeBatchEffect” embedded in limma R package to obtain an adjusted dataset (Chua et al. 2024; Ritchie et al. 2015).

### 2.6 Gene Ontology (GO) Term Networking Analysis

R package clusterProfiler (Yu et al. 2012) (v.4.7.1) was utilized to perform functional enrichment analysis of significant module genes. The enrichGO function from the clusterProfiler package was utilized to analyze the GO biological process and Kyoto Encyclopedia of Genes and Genomes (KEGG) (Kanehisa et al. 2017) pathway enrichment of hub genes. The top ten GO terms with *p* value < 0.001 were defined as significant ones for each category. The connection was quantified using the pairwise_termsim function from the clusterProfiler package, which quantifies semantic similarity between GO terms based on gene overlap.

### 2.7 Strains

All *C. elegans* strains were maintained at 20°C on standard nematode growth medium (NGM) plates seeded with OP50 *E. coli* bacteria (Kim, Moon, Heo et al. 2024). Bristol strain N2 was used as wild‐type (WT).

### 2.8 Food Occupancy Assays

The assays were performed as described with minor modifications (Gaglia et al. 2012). NGM plates were supplemented with either 0.35% dimethyl sulfoxide (DMSO, Sigma‐Aldrich, MO, USA; control), 50 μM rifampicin (Sigma‐Aldrich, MO, USA), or 50 μM atracurium (Sigma‐Aldrich, MO, USA). Approximately 30 prefertile animals were transferred onto NGM plates containing DMSO, rifampicin, or atracurium. NGM plates were seeded with 10 μL of cultured OP50. The number of animals on the plates was counted 6 h after transfer. Images of the plates were captured using a DIMIS‐M (Siwon Optical Technology, South Korea) camera.

### 2.9 Feeding Assays

Pharyngeal pumping (feeding) rates of the animals were measured as described previously (Park et al. 2021), with minor modifications. Ten L4 larvae or prefertile adults were grown on OP50‐seeded NGM plates supplemented with 0.35% DMSO, 50 μM rifampicin, or 50 μM atracurium. For fed conditions, animals were transferred to fresh OP50‐seeded NGM plates containing the same compounds. For dietary restriction (DR) conditions, animals were transferred to unseeded NGM plates containing the same compounds and supplemented with 100 μg/mL ampicillin (USB, Santa Clara, CA, USA) to prevent bacterial growth. After 6 h of incubation, the number of pumpings was counted for 30 s by observing the pharyngeal pumping of an animal under a dissecting microscope, and the measurements were rescaled to the number of pumpings per min.

### 2.10 Lifespan Assays

Lifespan assays were performed as previously described (Park et al. 2021), with minor modifications. Briefly, age‐synchronized *C. elegans* larvae were cultured on OP50‐seeded NGM plates supplemented with either 0.35% DMSO, 50 μM rifampicin, or 50 μM atracurium until the animals reached the prefertile young adult stage at 20°C. The animals were then transferred to the indicated NGM plates, supplemented with either DMSO or rifampicin, containing 5 μM 5‐fluoro‐2′‐deoxyuridine (FUDR, Sigma‐Aldrich, MO, USA) to prevent progeny from hatching. After 2 days, the animals were transferred to either OP50‐seeded NGM plates for fed conditions or NGM plates containing 100 μg/mL ampicillin to prevent bacterial growth for DR conditions. The animals were counted as dead if they did not respond to a gentle touch using a platinum wire. Animals that crawled off the plates, ruptured, displayed internal hatching, or burrowed were censored but included in the statistical analysis. Lifespan assays were performed by at least two independent researchers for reproducibility and analyzed individually. Statistical analysis was performed using a log‐rank (Mantel‐Cox method) test in OASIS2 (https://sbi.postech.ac.kr/oasis2) and OASIS portable (http://sbi.postech.ac.kr/oasis) (Han et al. 2024, 2016).

### 2.11 Measurement of Brood Size and Progeny Profile

A single L4 stage animal was transferred onto an NGM plate supplemented with either 0.35% DMSO, 50 μM rifampicin, or atracurium. For fed conditions, the plates were seeded with OP50, whereas for DR conditions, plates were left unseeded. Each animal was transferred onto a new NGM plate supplemented with either 0.35% DMSO, 50 μM rifampicin, or 50 μM atracurium every 12 h until it stopped laying eggs for two consecutive days. The number of viable larvae descended from a single animal was counted as the brood size. The average of the progeny number curves for each time point was considered as the progeny profile. The sum of the progeny numbers for all the counted days was considered as the total brood size. For each condition, 20 animals were counted for brood size. Animals that died, displayed internal hatching, or crawled off before stopping laying eggs were excluded from the analysis. A two‐tailed Student's *t* test was used for statistical analysis.

## 3 Results

### 3.1 Development of T‐CLASS Computational Framework for Transcriptomic Classification

We developed T‐CLASS, a comprehensive computational framework to classify physiological states of interest, such as longevity and aging, using transcriptomic data as the input. We aimed to provide this as a one‐stop analytic platform that (1) integrates transcriptome profiles, (2) identifies optimal and minimal gene sets distinguishing different physiological states, (3) categorizes related transcriptional landscapes using these gene sets, and (4) classifies the physiological state of specific transcriptomes (Figure 1a; see Methods for details). T‐CLASS should enable rapid and intuitive interpretation of expression patterns that simplify explorative transcriptomic data analysis, facilitating hypothesis generation for further experimentation. The input into T‐CLASS is the query and the reference transcriptome data in the form of the count matrix from RNA‐seq, labeled with physiological states.

T‐CLASS provides key statistical procedures and outputs for understanding transcriptomic differences associated with specific physiological states. The outputs are (1) optimal gene set, (2) similarities between query and categories, and (3) visualization of expression patterns of optimal gene sets (Figure 1b). T‐CLASS employs ANOVA to rank genes based on their contributions to distinguishing physiological states and subsequently uses the elbow method to determine the optimal number of top‐ranked genes. The resulting gene set is a T‐CLASS output where the gene expression patterns are not confounded by technical or biological factors unrelated to the physiological states of interest. By performing dimensional reduction on this selected gene set, T‐CLASS identifies coordinates that most effectively capture subtle, state‐specific transcriptomic differences. T‐CLASS visualizes these differences with heatmaps to provide an overview of underlying expression patterns. Consequently, researchers can evaluate the degree of physiological differences among samples from transcriptomic perspectives. T‐CLASS is accessible via web browsers (Figure 1c; http://www.t‐class.kaist.ac.kr).

### 3.2 T‐CLASS Faithfully Detects Transcriptome Changes Among Aging States

As the first test case for determining the usefulness of T‐CLASS for aging research, we sought to classify various biological contexts in longevity‐promoting regimens in the nematode *C. elegans* . The established longevity‐promoting regimens in *C. elegans* that we selected were rIIS, DR, and rMF (Lee et al. 2015). We performed transcriptome profiling and data integration of 27 publicly available RNA‐seq datasets of long‐lived *C. elegans* mutants in the aforementioned three representative longevity‐promoting regimens (Figure S1a; See Table S1 for detailed GEO accession numbers of the analyzed datasets). We then sought to establish a statistically rigorous transcriptomic comparison that identifies differences in gene expression resulting from the genetic interventions that promote longevity by using T‐CLASS.

**FIGURE 2:**
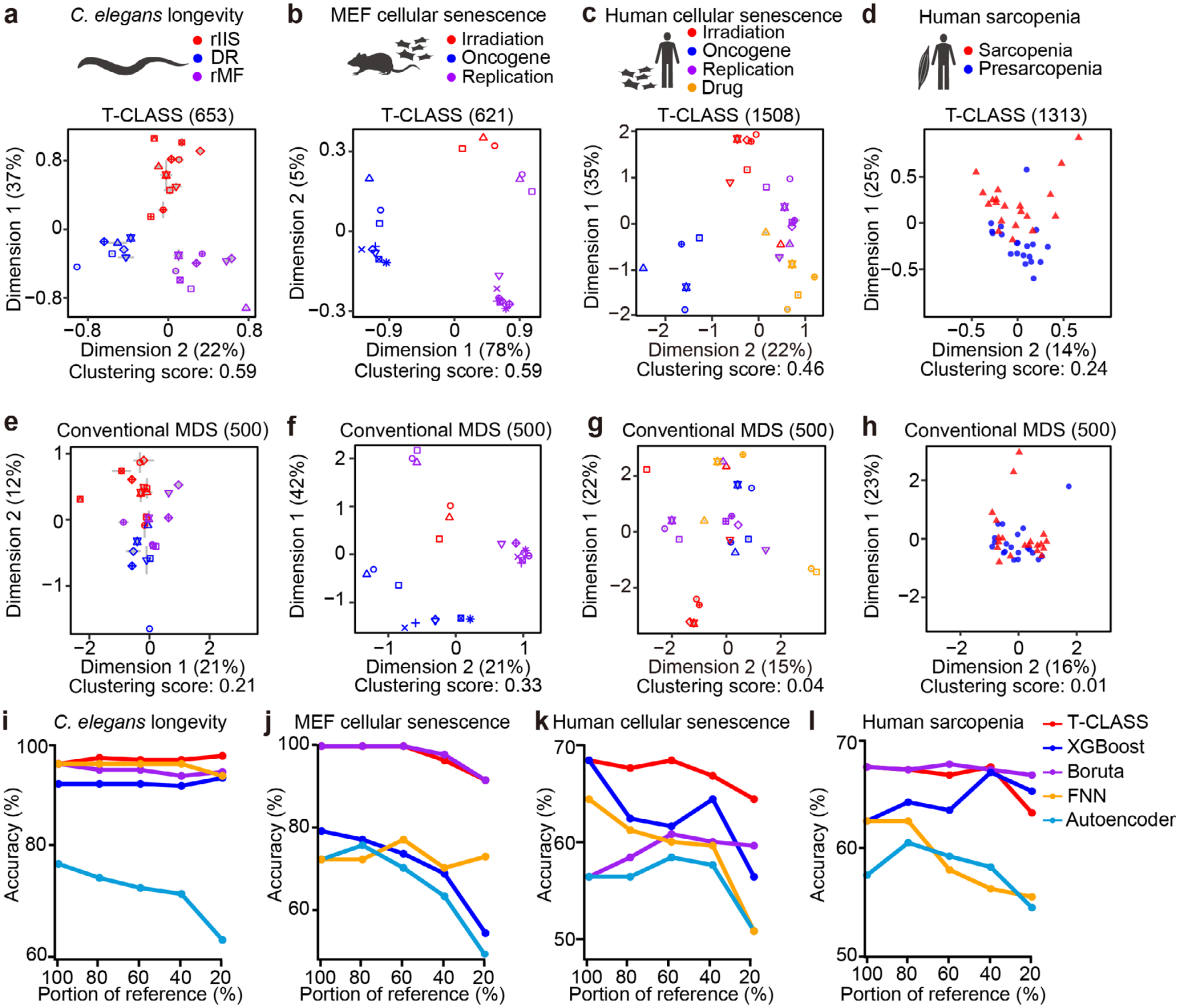
Transcriptome landscapes using T‐CLASS and conventional MDS in longevity‐promoting regimens in *Caenorhabditis elegans* , cellular senescence in both cultured mouse embryonic fibroblasts (MEF) and cultured human cells, and human sarcopenia. The performance of categorization was evaluated by calculating the clustering score (silhouette coefficient), comparing the results obtained using T‐CLASS and conventional MDS. (a, e) MDS plots of the transcriptomic changes caused by three longevity‐promoting regimens analyzed in *C. elegans* : reduced insulin/IGF‐1 signaling pathway (rIIS, red), dietary restriction (DR, blue), and reduced mitochondrial function (rMF, purple) by using T‐CLASS (653 genes) (a) and conventional MDS (500 genes) (e). (b, f) MDS plots of transcriptomic changes caused by cellular senescence inducers in MEF (Kang et al. 2024; Lozano‐Torres et al. 2019): Irradiation (red), oncogene‐induced senescence (blue), and extensive replication (purple) by using T‐CLASS (621 genes) (b) and conventional MDS (500 genes) (f). (c, g) MDS plots of transcriptomic changes caused by cellular senescence inducers in cultured human cells: Irradiation (red), oncogene (blue), extensive replication (purple), and drugs (orange) by using T‐CLASS (1508 genes) (c) and conventional MDS (500 genes) (g). (d, h) MDS plots of transcriptomic changes caused by human sarcopenia: sarcopenia (red) and presarcopenia (blue) by using T‐CLASS (1313 genes) (d) and conventional MDS (500 genes) (h). See Figure S2 for the transcriptome landscapes using machine/deep learning‐based tools including Extreme Gradient Boosting (XGBoost), Boruta, feedforward neural network (FNN), and autoencoder using four transcriptomic datasets: longevity‐promoting regimens in *C. elegans* , cellular senescence in mouse embryonic fibroblasts and cultured human cells, and human sarcopenia. (i–l) Evaluation of classification accuracy using leave‐one‐out cross‐validation across varying proportions of reference data of five gene selection methods, T‐CLASS (red), XGBoost (blue), Boruta (purple), feedforward neural network (FNN) (orange), and autoencoder (sky blue), in four transcriptomic datasets: *C. elegans* longevity (i), MEF cellular senescence (j), human cellular senescence (k), and human sarcopenia (l). In leave‐one‐out cross‐validation, each dataset (or sample) was used as the query, while the remaining datasets were used as the reference to assess classification performance. To assess the robustness of classification, 100%, 80%, 60%, 40%, and 20% of the reference datasets were randomly sampled, and 10‐fold cross‐validation was performed. See Table S1 for detailed GEO accession numbers of the analyzed datasets.

T‐CLASS identified 653 genes as the optimal gene set for distinguishing rIIS, DR, and rMF (Figure S1b). We showed that the 27 datasets were distinctively grouped into the three established longevity‐promoting regimens when using the optimal gene set of the 653 genes (Figure 2a; clustering score: 0.59) compared to the default option for gene selection in MDS packages (Figure 2e; clustering score: 0.21). These results demonstrate the ability of T‐CLASS in robustly classifying transcriptomic signatures associated with a complex phenotype, namely animal longevity.

Next, we validated the utility of T‐CLASS by analyzing public RNA‐seq datasets with cellular senescence in mouse embryonic fibroblasts (MEF) (Figure S1c; See Table S1 for detailed GEO accession numbers of the analyzed datasets) and in cultured human cells (Figure S1e; See Table S1 for detailed GEO accession numbers of the analyzed datasets). The inducers of cellular senescence in MEF and in cultured human cells were irradiation, oncogene activation, extensive replication, and drug (Kang et al. 2024; Lozano‐Torres et al. 2019; Yan et al. 2024). T‐CLASS identified the optimal 621 genes for cellular senescence in MEF (Figure S1d) and the optimal 1508 genes for human cellular senescence (Figure S1f). By using these optimal gene numbers, T‐CLASS effectively distinguished the transcriptomic changes caused by different types of cellular senescence in MEF and human (Figure 2b,c; clustering score: 0.59 and 0.46) compared to conventional MDS (Figure 2f,g; clustering score: 0.33 and 0.04). These findings indicate the versatility of T‐CLASS in categorizing transcriptomic signatures across different biological contexts.

We also validated the effectiveness of T‐CLASS by using human sarcopenia datasets (Figure S1g; See Table S1 for detailed GEO accession numbers of the analyzed datasets). T‐CLASS identified the optimal 1313 genes for human sarcopenia (Figure S1h). By using these genes, T‐CLASS successfully distinguished the transcriptome changes of sarcopenia and presarcopenia patients (Figure 2d; clustering score: 0.24) compared to conventional MDS (Figure 2h; clustering score: 0.01).

We next compared the performance of T‐CLASS with four additional gene selection methods: (i) Extreme Gradient Boosting (XGBoost) (Chen and Guestrin 2016), (ii) machine learning‐based approach Boruta (Kursa 2014), (iii) feedforward neural network (FNN) (Candel et al. 2016), and (iv) autoencoder (Gulli and Pal 2017) (Figure 2i–l and Figure S2). XGBoost and Boruta are machine learning‐based gene selection tools that use ensemble learning of decision trees, with Boruta offering robust feature selection against overfitting. FNN and autoencoder are deep learning‐based methods; FNN selects genes by minimizing classification loss, while the autoencoder identifies informative genes through unsupervised reconstruction of input data. We evaluated the classification performance of each method using leave‐one‐out cross‐validation (Xu and Liang 2001), which assesses classification accuracy by using a single dataset (or sample) as the query, the remaining data as the reference, and measuring how accurately the query is classified. We also assessed the stability of classification accuracy when using all or subsets of the reference datasets at varying proportions (Figure 2i–l). Overall, T‐CLASS exhibited consistently high and robust classification accuracy across datasets, particularly for *C. elegans* longevity and human cellular senescence (Figure 2i,k). In contrast, Boruta showed comparable or better performance compared to T‐CLASS for analyzing datasets that are composed of individual biological replicates, in which cases complex batch effects arise (Yu et al. 2024), such as mouse cellular senescence (Figure 2j) and human sarcopenia (Figure 2l). These results indicate that T‐CLASS generally outperforms other tools, while Boruta's robust gene selection provides advantages in challenging situations where batch correction is critical. To accommodate varying data characteristics, our T‐CLASS web platform provides Boruta as well, allowing users to select the most suitable option for their dataset.

### 3.3 The *C. elegans* Optimal Gene Set Contains Biological Information Specific for the Different Longevity‐Promoting Regimens

To determine the effectiveness of T‐CLASS in distinguishing the transcriptomic changes in more depth, we focused our further analyses on transcriptomic changes associated with *C. elegans* longevity. We noticed a strong positive correlation between the Euclidean distance of each sample from the control and the extent of lifespan extension by rIIS (Figure S3a,b). rIIS conditions that displayed relatively large lifespan‐extending effects, including *daf‐2(e1370)* mutants and DAF‐2 auxin‐inducible protein degradation (AID) strain (Kwon et al. 2023; Lee et al. 2015), elicited greater Euclidean distances from the control compared to conditions with relatively small lifespan‐extending effects: *daf‐2(e1368)* mutants and *daf‐2* RNAi‐treated animals (Figure S3b). In contrast, DR or rMF regimens did not exhibit significant correlation between the Euclidean distance from the control and the extent of lifespan (Figure S3c,d). These data imply that T‐CLASS can be used to predict the strength of an allele on longevity and aging processes, such as *daf‐2* variants on lifespan extension, but this is not generalizable.

**FIGURE 3:**
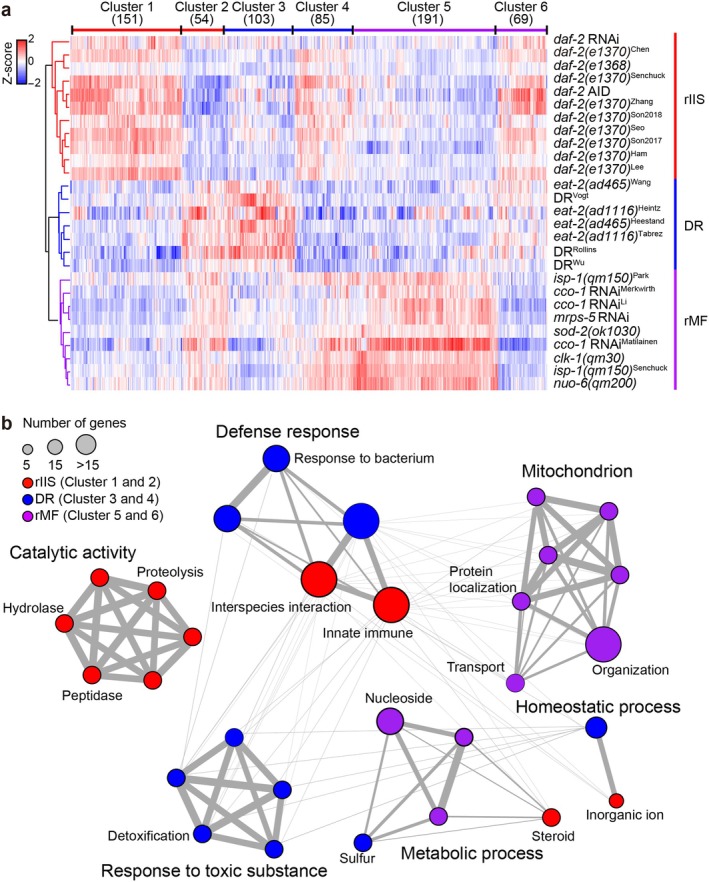
Analysis of the 653 optimal genes used for the classification of *C. elegans* longevity. (a) A clustering heatmap of the 653 genes in long‐lived mutants and conditions: rIIS, DR, and rMF. Count values were transformed by using variance stabilizing transformation, and were normalized into Z‐score. Hierarchical clustering was performed based on Euclidian distances to group samples. References for datasets of the same mutant alleles or conditions are indicated as superscripts next to each mutant allele or condition. (b) Gene ontology (GO) enrichment analysis of the 653 genes by using R package clusterProfiler (Yu et al. 2012). Top 10 GO terms with *p* value < 0.001 were defined as significant ones for each category. Node size represents the number of genes in each term. Edge thickness represents the semantic similarity between the connected GO terms, which is determined by the number of shared genes and their relative hierarchical position in the GO network.

We then asked how the optimal 653 gene set distinguished the three longevity‐promoting regimens in *C. elegans* . By comparing gene expression patterns of the 27 longevity conditions that we obtained, samples within each regimen displayed similar expression patterns of the 653 genes, demonstrating regimen‐specific gene expression (Figure 3a; Table S3). This was confirmed by clustering analysis (Figure 3a). Here, the 653 optimal gene set was divided into six clusters with distinct expression patterns (Table S4): cluster 1 with higher expression in rIIS than in DR and rMF (151 genes), cluster 2 with lower expression in rIIS than in DR and rMF (54 genes), cluster 3 with higher expression in DR than in rIIS and rMF (103 genes), cluster 4 with lower expression in DR than in rIIS and rMF (85 genes), cluster 5 with higher expression in rMF than in rIIS and DR (191 genes), and cluster 6 with lower expression in rMF than in rIIS and DR (69 genes).

We next determined whether the genes within the six clusters are implicated in distinct biological functions by performing GO enrichment analysis, and functional enrichment analysis using WormCat (Higgins et al. 2022; Holdorf et al. 2020) and KEGG (Figure 3b and Figure S4). We identified the GO term “Defense response,” an established and crucial term for longevity conferred by rIIS and DR (Lee et al. 2021; Naim et al. 2021; Wu et al. 2019). For the rIIS‐specific GO terms, we identified “Catalytic activity,” including “Peptidase,” as an rIIS‐specific term, raising the possible role of the activity of insulin‐degrading enzyme (IDE), an endopeptidase known to regulate insulin metabolism (Hong et al. 2008), in rIIS‐mediated longevity. Identification of DR‐specific GO terms, such as “Response to toxic substance,” is consistent with the finding that DR enhances detoxification in rats as well as in *C. elegans* (Cypser et al. 2013; Wen et al. 2013). As expected, we found that various “Mitochondrion” terms were enriched in rMF‐specific terms. By performing functional enrichment analysis using WormCat, KEGG pathway enrichment, and GO analysis for Cellular Component and Molecular Function, we also identified terms and pathways that were similar to those shown in Figure 3b (Figure S4). Together, these data demonstrate that the 653 genes effectively represent specific as well as common biological processes associated with the three longevity regimens.

### 3.4 T‐CLASS Successfully Classifies Mutants to Their Corresponding Longevity‐Promoting Regimen

**FIGURE 4:**
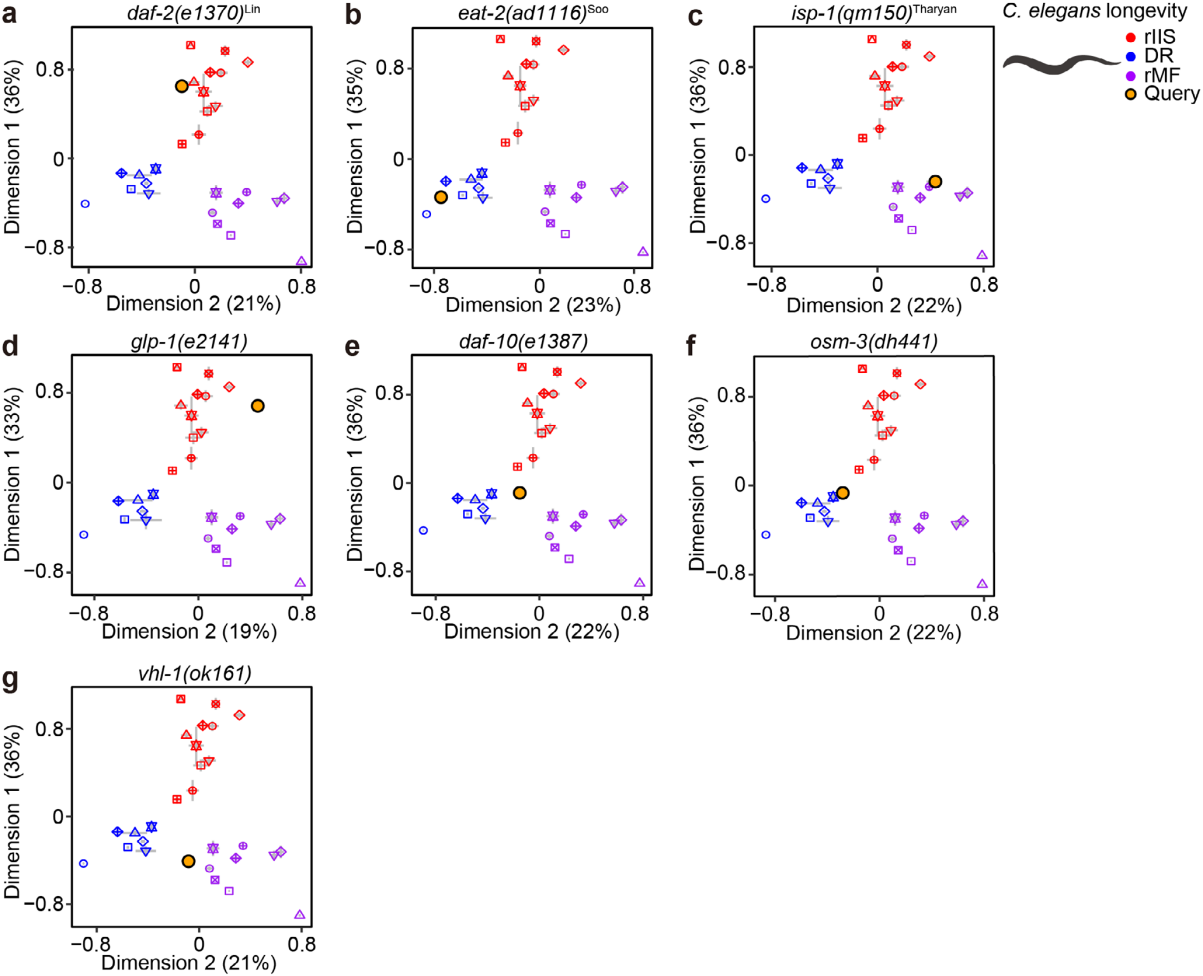
Validation and classification of long‐lived mutants into the three longevity‐promoting regimens using T‐CLASS for *C. elegans* dataset. (a–c) Validation of classification by T‐CLASS by using established long‐lived mutants in each of the three longevity‐promoting regimens: *daf‐2(e1370)* ^Lin^ (Lin et al. 2018) in rIIS (a), *eat‐2(ad1116)* ^Soo^ (Soo et al. 2023) in DR (b), and *isp‐1(qm150)* ^Tharyan^ (Tharyan et al. 2020) in rMF (c). References for datasets are indicated as superscripts next to each mutant allele. (d–g) Classification of four long‐lived mutants with ambiguous association with the three longevity‐promoting regimens: *glp‐1(e2141)* (GEO accession: GSE193444) (d), *daf‐10(e1387)* (Li et al. 2024) (e), *osm‐3(dh441)* (Guerrero et al. 2021) (f), and *vhl‐1(ok161)* (Ganner et al. 2021) (g). Error bars indicate standard error of mean (SEM). Yellow dot indicates query in each of MDS plots.

The next step we took in the validation of T‐CLASS was to test whether established longevity mutants, when their data were entered as a query, were classified to the corresponding longevity‐promoting regimen: *daf‐2* mutant for rIIS, *eat‐2* mutant for DR, and *isp‐1* mutant for rMF (Lee et al. 2015). By using Spearman correlation, we showed that the aforementioned mutants were classified to the associated longevity regimens (Figure 4a–c; Table S5). We then tested whether T‐CLASS can be utilized for classifying long‐lived mutants with ambiguous associations with any of the three longevity‐promoting regimens (Figure 4d–g). We used transcriptome data of long‐lived *glp‐1*, *daf‐10*, *osm‐3*, and *vhl‐1* mutants (Li et al. 2024; Guerrero et al. 2021; Ganner et al. 2021; See Table S1 for detailed GEO accession numbers of the analyzed datasets). We found that transcriptomic changes resulting from the *glp‐1* mutation, which causes germline deficiency to promote longevity (Hsin and Kenyon 1999), were closely located to rIIS on the MDS plot (Figure 4d; Table S5). This is consistent with previous findings showing that the *glp‐1* mutation shares components with rIIS for the activation of DAF‐16/FOXO, a transcription factor critical for longevity (Hsin and Kenyon 1999). Mutations in *daf‐10* and *osm‐3* cause defects in sensory neurons, which are critical for sensing diet‐derived cues and for regulating aging by acting upstream of IIS (Alcedo and Kenyon 2004; Artan et al. 2016; Smith et al. 2008). Thus, *daf‐10* and *osm‐3* mutants were expected to be classified as either DR or rIIS. Indeed, we found that transcriptome changes caused by *daf‐10* mutations were located between DR and rIIS on the MDS plot (Figure 4e; Table S5), whereas *osm‐3* mutations were classified as DR (Figure 4f; Table S5). We further deconvoluted the mixed effect caused by the *daf‐10* mutation and found that the transcriptome changes in the *daf‐10* mutant were more similar to DR than to rIIS (Figure S5a,b). Thus, sensory deprivation appears to cause transcriptome changes similar to those induced by DR. The *vhl‐1* mutation that causes hyperactivation of hypoxia‐inducible factor 1 (HIF‐1) is known to act together with rMF, by regulating common downstream effectors (Hwang and Lee 2011; Lee et al. 2010; Leiser and Kaeberlein 2010). Consistently, transcriptomic changes caused by the *vhl‐1* mutation were classified as rMF (Figure 4g; Table S5). Thus, T‐CLASS successfully classified these test case longevity mutations into the specific longevity‐promoting regimens, demonstrating the potential of T‐CLASS to uncover new biological insights of the aging‐regulatory paradigm from transcriptome data.

### 3.5 T‐CLASS Can Properly Classify Lifespan‐Extending Small Molecules

**FIGURE 5:**
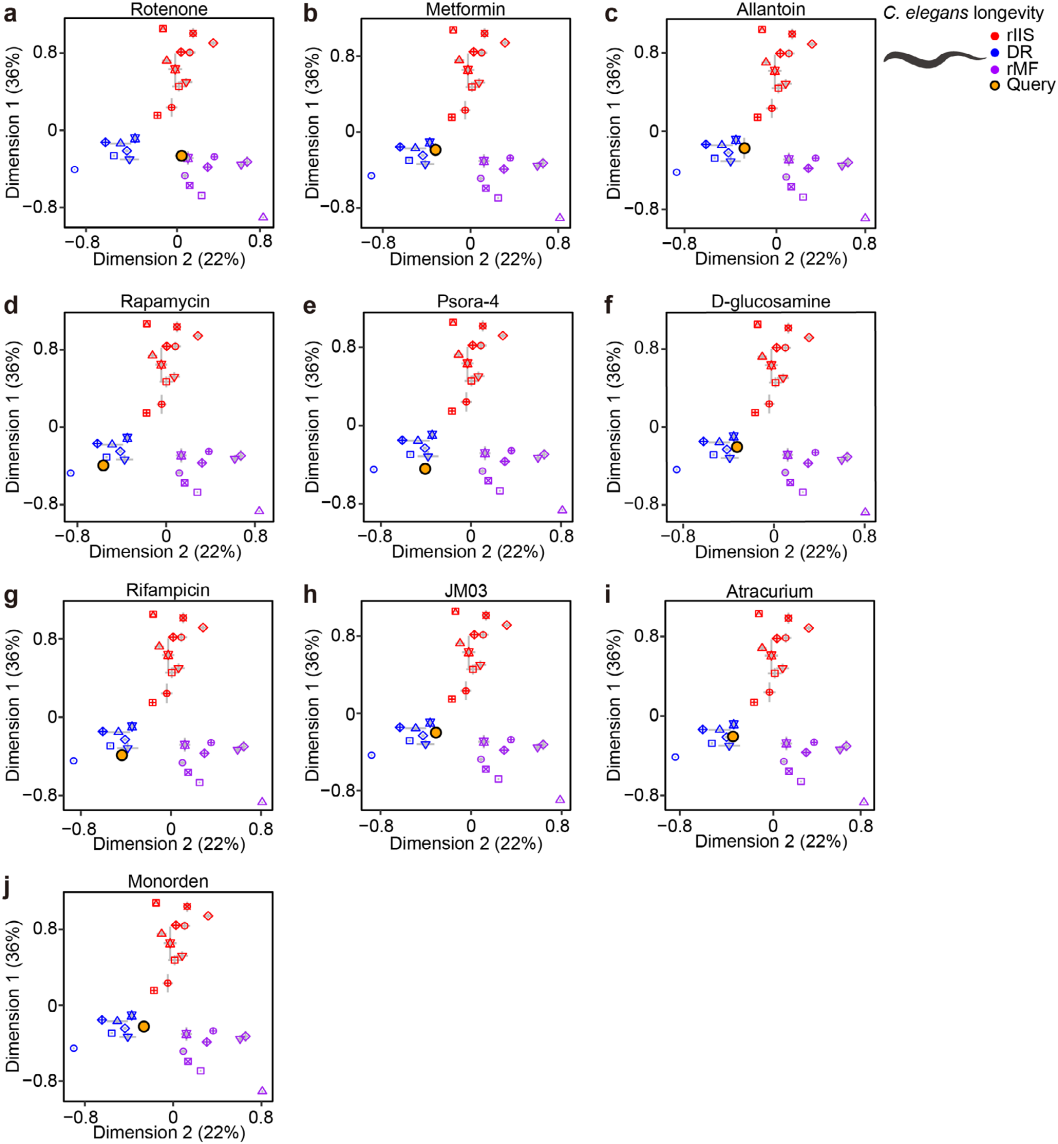
Classification of ten longevity‐promoting small molecules into the three longevity‐promoting regimens by using T‐CLASS. (a–j) Classification of each of the ten longevity‐promoting small molecules, including rotenone (Mello et al. 2022) (a), metformin (Admasu et al. 2018) (b), allantoin (Admasu et al. 2018) (c), rapamycin (Admasu et al. 2018) (d), psora‐4 (Admasu et al. 2018) (e), D‐glucosamine (Weimer et al. 2014) (f), rifampicin (Admasu et al. 2018) (g), JM03 (Bao et al. 2022) (h), atracurium (McIntyre et al. 2021) (i), and monorden (Janssens et al. 2019) (j). Error bars indicate standard error of mean (SEM). Yellow dot indicates query in each of MDS plots.

Next, we sought to classify longevity‐promoting small molecules using T‐CLASS. We analyzed the transcriptomic changes caused by ten longevity‐promoting small molecules: rotenone, metformin, allantoin, rapamycin, psora‐4, D‐glucosamine, rifampicin, JM03, atracurium, and monorden (Admasu et al. 2018; Bao et al. 2022; Janssens et al. 2019; McIntyre et al. 2021; Mello et al. 2022; Weimer et al. 2014). As expected, T‐CLASS classified rotenone, a mitochondrial electron transport chain complex inhibitor, to rMF (Figure 5a; Table S5). We also found that five small molecules that have been established to promote longevity in a DR‐like manner in *C. elegans* (Admasu et al. 2022; Calvert et al. 2016; Kumar et al. 2021; Onken and Driscoll 2010), metformin, allantoin, rapamycin, psora‐4, and D‐glucosamine, were classified as DR (Figure 5b–f; Table S5). These data indicate that T‐CLASS accurately categorizes the query data obtained from treatment with the small molecules. Importantly, rifampicin, JM03, atracurium, and monorden, which were not previously categorized, were classified as DR (Figure 5g–j; Table S5), suggesting that these four small molecules confer longevity by mimicking DR. We hence set out to experimentally validate this finding for rifampicin and atracurium.

### 3.6 Rifampicin and Atracurium Promote Longevity via DR

**FIGURE 6:**
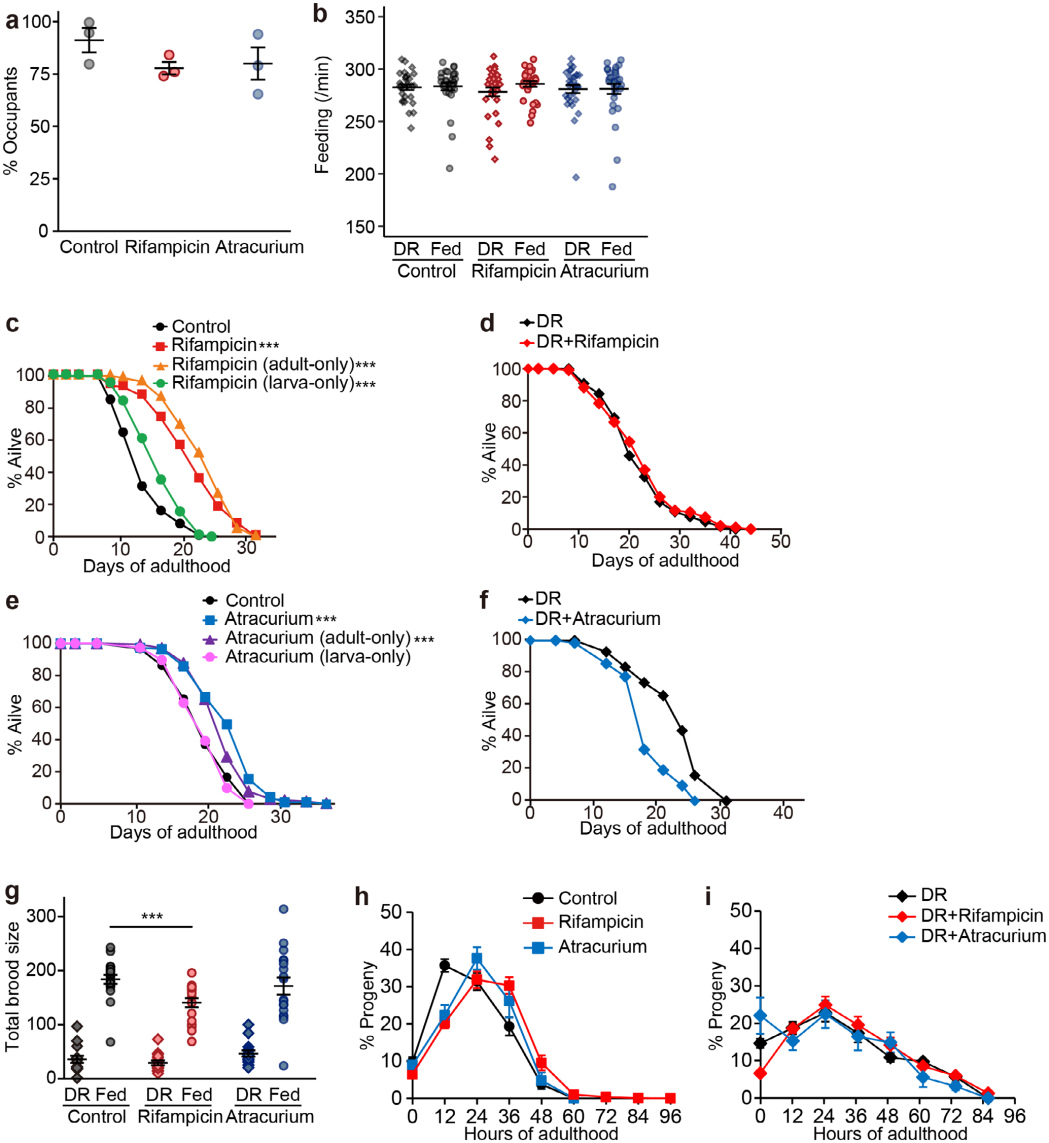
Rifampicin and atracurium promote longevity similarly to DR. (a) Food occupancy was assessed on *E. coli* OP50‐seeded plates supplemented with either DMSO (control), rifampicin, or atracurium. The % animals that occupied (% occupants) the bacterial lawn was quantified (*n* = 3). Error bars represent standard error of mean (SEM). (b) Pharyngeal pumping (feeding) rates of prefertile animals treated with rifampicin and atracurium compared to control (*N* = 10 for each condition, pharyngeal pumping per minute for three independent trials). Error bars represent SEM. (c) Rifampicin substantially increased the lifespan of DMSO‐treated (control) animals (54.7%, *p* < 0.001). Treatment with rifampicin during adult stage (adult‐only) robustly increased lifespan (68%, *p* < 0.001) but that during larval stage (larva‐only) had small effects (17.3%, *p* < 0.001). (d) Rifampicin did not further increase lifespan under DR conditions (1.7%, *p* = 0.5). (e) Atracurium increased the lifespan of DMSO‐treated (control) animals (17.1%, *p* < 0.001). Adult‐only atracurium treatment significantly increased lifespan (13.6%, *p* < 0.001), whereas larval‐only treatment had no effect (−0.4%, *p* = 0.6). (f) Atracurium did not further increase lifespan under DR conditions (−18.8%, *p* < 0.001). (g) Total brood size of animals treated with rifampicin or atracurium compared to control. (h, i) Progeny profiles of rifampicin‐ or atracurium‐treated animals compared to control animals under fed (h) and DR (i) conditions. Asterisks in the survival curve panels indicate the significance of differences (***p < 0.001), calculated using a log‐rank (Mantel‐Cox) test. See Table S6 for additional repeats and statistical analysis.

We chose to test whether treatment with rifampicin and atracurium caused physiological changes similar to DR for the following reasons. Rifampicin and atracurium were among the four DR‐classified small molecules (rifampicin, JM03, atracurium, and monorden) whose association with DR was uncertain (Figure 5g–j; Table S5). However, a previous study raised the possibility that rifampicin actually alters the structure of mitochondria, leading to dysfunction (Erokhina et al. 2013). In addition, atracurium may extend healthspan differently from DR because atracurium did not affect pharyngeal pumping rate or body size in *C. elegans* (McIntyre et al. 2021). Thus, we experimentally tested the association of rifampicin‐ and atracurium‐mediated longevity with DR. We first sought to test the possibility that rifampicin and atracurium simply induced DR in *C. elegans* by reducing food occupancy or feeding rates. As we did not observe any differences to control treatment, rifampicin and atracurium are unlikely to directly limit feeding (Figure 6a,b). We showed that treatment with rifampicin and atracurium during adult stage (adult‐only) increased lifespan but that during larval development (larva‐only) had a small effect (Figure 6c,e); this is consistent with established findings that the longevity‐promoting effects of DR act at the adult stage (Lee et al. 2006), while being different from rMF‐mediated longevity that acts during larval development (Dillin et al. 2002). In addition, we found that rifampicin and atracurium did not substantially increase the long lifespan of animals on DR (Figure 6d,f; Table S6), suggesting that rifampicin and atracurium promote longevity through the same pathway as DR. Rifampicin and atracurium reduced early progeny production under fed conditions but increased late progeny production, another key characteristic of *C. elegans* DR (Angelo and Van Gilst 2009; Scharf et al. 2021), and had little or no effect on total brood size (Figure 6g,h). Notably, under DR conditions, treatment with either drug did not induce a further shift compared to DR control (Figure 6i), consistent with their classification as DR‐mimetic compounds. Overall, these data indicate that rifampicin and atracurium extend lifespan in a way similar to DR, validating our T‐CLASS for accurately classifying longevity‐promoting small molecules.

## 4 Discussion

Investigating how physiological changes affect the transcriptome is crucial for understanding molecular mechanisms underlying longevity and aging. In this study, we developed T‐CLASS, an intuitive transcriptome‐based classification online tool for identifying gene sets that most effectively interpret changes in physiological states for the iterative classification of physiological and cellular aging processes. We evaluated the effectiveness of T‐CLASS using transcriptomes, including longevity‐promoting regimens in the nematode, senescent cells in both mice and humans, and in human sarcopenia. We also found that the classification accuracy of T‐CLASS was consistently high and robust compared to other machine/deep learning tools. As proof of principle, we analyzed the effect of rifampicin and atracurium on longevity by using T‐CLASS, which classified the two drugs as DR, and validated the results by performing in vivo physiological experiments using *C. elegans* . T‐CLASS will be a highly useful tool for researchers in the aging research field working with transcriptome data to specify functional effectors of various physiological categories and will accelerate deciphering underlying molecular mechanisms.

Selecting a classification method that matches the specific characteristics of a dataset is crucial for accurate performance. Specifically, sample‐level analysis (i.e., using individual biological replicates) is more sensitive to batch effects than dataset‐level analysis (i.e., using mean expression values across replicates) (Yu et al. 2024). Our results showed that T‐CLASS and Boruta performed best in dataset‐level and sample‐level analyses, respectively (Figure 2i–l), highlighting the importance of properly choosing methods that can effectively address such variability. Therefore, providing multiple analysis options may improve flexibility and accuracy in transcriptomic classification, depending on dataset structure and study objectives.

MDS has advantages of compressing and projecting similarities among clusters onto two dimensions with minimum distortion of the original pairwise distances or dissimilarities. However, conventional MDS causes challenges in effectively detecting differences in transcriptome among groups because of the inclusion of nonessential information and the curse of dimensionality (Altman and Krzywinski 2018; Lin and Fukuyama 2024). By applying basic parametric statistics that capture gene set with linear increase and decrease in gene expression, MDS can more accurately project transcriptional similarities among groups. Superior performance of T‐CLASS in projection compared to conventional MDS approaches will lessen the burden for researchers who seek proper classification and interpretation of transcriptome data. Additionally, compared with machine learning‐based approaches, such as XGBoost, Boruta, FNN, and autoencoder, which produce different sets of genes in runs because of their inherent randomness (Table S2), T‐CLASS consistently generates stable sets of optimal genes, enabling subsequent analyses, such as generation of heatmaps for overall expression patterns and GO analysis. Other tools for categorizing and projecting transcriptome data onto two dimensions are also available. For example, Seurat, one of the most widely used tools for single‐cell RNA sequencing (scRNA‐seq) analysis, provides unsupervised algorithms for categorization (Satija et al. 2015). However, Seurat failed to achieve effective categorization compared to T‐CLASS (Figure S6), likely because Seurat is useful for handling scRNA‐seq datasets with a large number of cells, while being suboptimal for moderate amounts of bulk RNA‐seq datasets. Overall, T‐CLASS provides users with optimal gene sets, offering a straightforward and intuitive interpretation for RNA‐seq data analysis compared to other well‐established tools including XGBoost, Boruta, FNN, autoencoder, and Seurat.

The classification of four longevity‐promoting small molecules (rifampicin, JM03, atracurium, and monorden) into DR by T‐CLASS raises two possible explanations. First, these chemicals may affect the expression and/or the activity of factors crucial for DR‐induced longevity. Second, *C. elegans* are known to exhibit attraction or avoidance behaviors in response to varying concentrations of chemicals (Choi et al. 2022), and therefore these chemicals may propel the animals from sufficient feeding. Our experimental data on rifampicin and atracurium align with the first possibility, due to the lack of obvious feeding defects in rifampicin and atracurium. Nevertheless, further research is required to precisely distinguish these possibilities and to elucidate underlying mechanisms guided by our T‐CLASS analysis.

Aging trajectories and physiological age are influenced by a variety of biological processes, including telomere dynamics (Projahn et al. 2024; Chevalier et al. 2025; Jäger et al. 2022; Gomes et al. 2011). Future research on the development of tools that incorporate key aging processes, such as telomere dynamics and tissue‐ or organ‐specific regulatory mechanisms, will be crucial for developing methods to accurately predict and classify biological age across species. In addition, developing classification methods that incorporate data beyond transcriptome, including methylation, will be crucial for advancing our understanding of longevity and aging.

Implementing transcriptomic data derived from the same tissue or cell line across samples will be ideal to avoid false discoveries when comparing biological conditions. Although T‐CLASS does not incorporate functions to explicitly remove tissue or cell line effects, our results indicate that it works reliably even when such confounding factors are present. For example, in a human cellular senescence dataset originated from multiple cell lines, T‐CLASS successfully classified various types of senescence inducers, including irradiation, oncogene, replication, and drug (Figure 2i–l). However, incorporating a new algorithm or method into T‐CLASS to control confounding effects could further improve the tool in future versions.

Integrating transcriptome data and performing downstream analyses using various organisms are crucial for deciphering evolutionarily conserved molecular pathways. T‐CLASS efficiently integrated and classified the transcriptomes associated with physiological changes, such as aging. The three longevity‐promoting regimens that we primarily focused on in our study are conserved across various species, including *C. elegans* , yeast, fruit fly, mouse, and human (C. Kenyon 2005; Kim and Lee 2019; Lee et al. 2015; Pan and Finkel 2017; Partridge et al. 2011; Zong et al. 2024). Given the universality of T‐CLASS for analyzing transcriptome data, future research can explore whether the transcriptome signatures associated with longevity and aging are conserved across different species. Our work also demonstrates that the transcriptomic state, which is classified by using the optimal gene set identified with T‐CLASS, can be a useful predictive biomarker for categorizing subtypes of biological processes, including diverse age‐associated disease conditions. In conclusion, T‐CLASS has the potential to serve as a powerful resource for expediting discovery and diagnosis based on transcriptomic data obtained in various organisms under diverse conditions.

## Author Contributions

S.‐C.J.L., G.‐Y.L., S.S.K., Y.B., S.H., S.K.H., and S.‐J.V.L. contributed to designing the research. S.‐C.J.L. and G.‐Y.L. performed data analyses. S.‐C.J.L., G.‐Y.L., Y.B., and S.H. performed data curation. S.‐C.J.L., G.‐Y.L., and S.K.H. implemented the web server. S.S.K., J.S., and G.‐Y.L. performed experiments. S.‐C.J.L., G.‐Y.L., S.S.K., S.K.H., and S.‐J.V.L. wrote the manuscript. All authors read and approved the manuscript.

## Conflicts of Interest

The authors declare no conflicts of interest.

## Supporting information


**Appendix S1:** acel70193‐sup‐0001‐AppendixS1.pdf.

**Appendix S2:** acel70193‐sup‐0002‐AppendixS2.pdf.

**Table S1:** acel70193‐sup‐0003‐TableS1.xlsx.

**Table S2:** acel70193‐sup‐0004‐TableS2.xlsx.

**Table S3:** acel70193‐sup‐0005‐TableS3.xlsx.

**Table S4:** acel70193‐sup‐0006‐TableS4.xlsx.

**Table S5:** acel70193‐sup‐0007‐TableS5.xlsx.

**Table S6:** acel70193‐sup‐0008‐TableS6.docx.

## Data Availability

The published sequencing data used in this study (Table S1) are available at Sequence Read Archive (SRA) and Gene Expression Omnibus (GEO) in National Center for Biotechnology Information (NCBI).
