# Supplementary material for: T‐CLASS: An Online Tool for the Identification and Classification of Aging and Senescence Using Transcriptome Data
Source: Aging Cell. 2025 Aug 14;24(10):e70193. doi: 10.1111/acel.70193 (PMC12507419; doi:10.1111/acel.70193)
Supplement: Supplementary file 1 — Appendix S1: acel70193‐sup‐0001‐AppendixS1.pdf. [file ACEL-24-e70193-s003.pdf]

Supplementary data

Supplementary figures

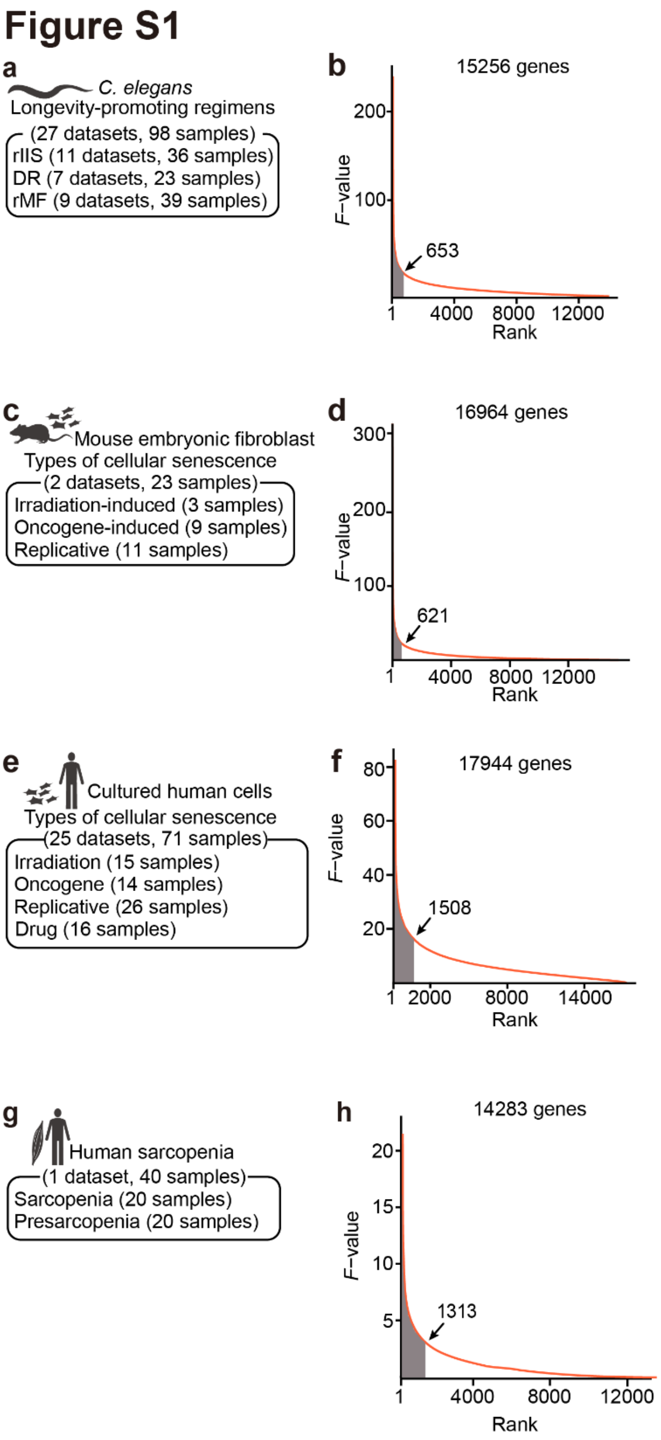

Figure S1. Selection of optimal gene sets for categorizing in multiple species and biological contexts. (**a**) Datasets analyzed for longevity-promoting regimens in *C.*

elegans: reduced insulin/IGF-1 signaling pathway (rIIS), dietary restriction (DR), and reduced mitochondrial function (rMF). For rIIS, we obtained eleven different datasets of long-lived *C. elegans* with decreased *daf-2*/insulin/IGF-1 signaling: eight *daf-2(e1370)* mutants (Chen et al., 2015; Ham et al., 2022; Y. Lee et al., 2021; Senchuk et al., 2018; Seo et al., 2015; Son et al., 2018; Son et al., 2017; Zhang et al., 2022), one *daf-2(e1368)* mutant (Chen et al., 2015), one *daf-2* RNAi-treated animals (Roitenberg et al., 2018), and one auxin-induced protein degradation (AID) strain targeting DAF-2 (Zhang et al., 2022). For DR, we analyzed eight datasets: three DR-mimetic *eat-2(ad1116)* mutants (Heintz et al., 2017; Tabrez et al., 2017), two DR-mimetic *eat-2(ad465)* mutants (Heestand et al., 2013; Wang et al., 2023), and three directly diet-restricted animals (Rollins et al., 2019; Vogt & Hobert, 2023; Wu et al., 2019). For rMF, we used nine datasets of mutants and RNAi-treated animals: two *isp-1* mutants (Park et al., 2021; Senchuk et al., 2018), one *clk-1* mutant (Senchuk et al., 2018), one *nuo-6* mutant (Senchuk et al., 2018), three *cco-1* RNAi-treated animals (Li et al., 2021; Matilainen et al., 2017; Merkwirth et al., 2016), one *mrps-5* RNAi-treated animals (Li et al., 2021), and one *sod-2* mutant (Senchuk et al., 2018). (**b**) Selection of optimal genes for categorizing the three longevity-promoting regimens in *C. elegans* by using the elbow method. The gene at rank 653 was the elbow point based on *F*-value. The 653 genes were then used as an optimal gene set for categorization. (**c**) Datasets analyzed for subtypes of cellular senescence in cultured mouse embryonic fibroblast: irradiation-induced, oncogene-induced, and replicative senescence (Gallage et al., 2024; Sturmlechner et al., 2022). (**d**) Selection of optimal genes for categorizing the subtypes of cellular senescence in cultured mouse embryonic fibroblast by using the elbow method. The gene at rank 621 was the elbow point based on *F*-value. Thus, the 621 genes were selected for

categorization. (**e**) Datasets analyzed for subtypes of cellular senescence in cultured human cells: irradiation-induced, oncogene-induced, replicative senescence, and drug (Marthandan et al., 2016; Casella et al., 2019; Yang et al., 2025; De Cecco et al., 2019; Joung et al., 2025; Cheng et al., 2024; Savić et al., 2023; Papaspyropoulos et al., 2023; Purcell et al., 2014) (**f**) Selection of optimal genes for categorizing the subtypes of cellular senescence in cultured human cells by using the elbow method. The gene at rank 1508 was the elbow point based on *F*-value. Thus, the 1508 genes were selected for categorization. (**g**) Datasets analyzed for human sarcopenia: patients with sarcopenia and presarcopenia (Zuo et al., 2025). (**h**) Selection of optimal genes for categorizing the subtypes of cellular senescence in cultured human cells by using the elbow method. The gene at rank 1313 was the elbow point based on *F*-value. Thus, the 1313 genes were selected for categorization. See Table S1 for detailed GEO accession numbers of the analyzed datasets.

Figure S2

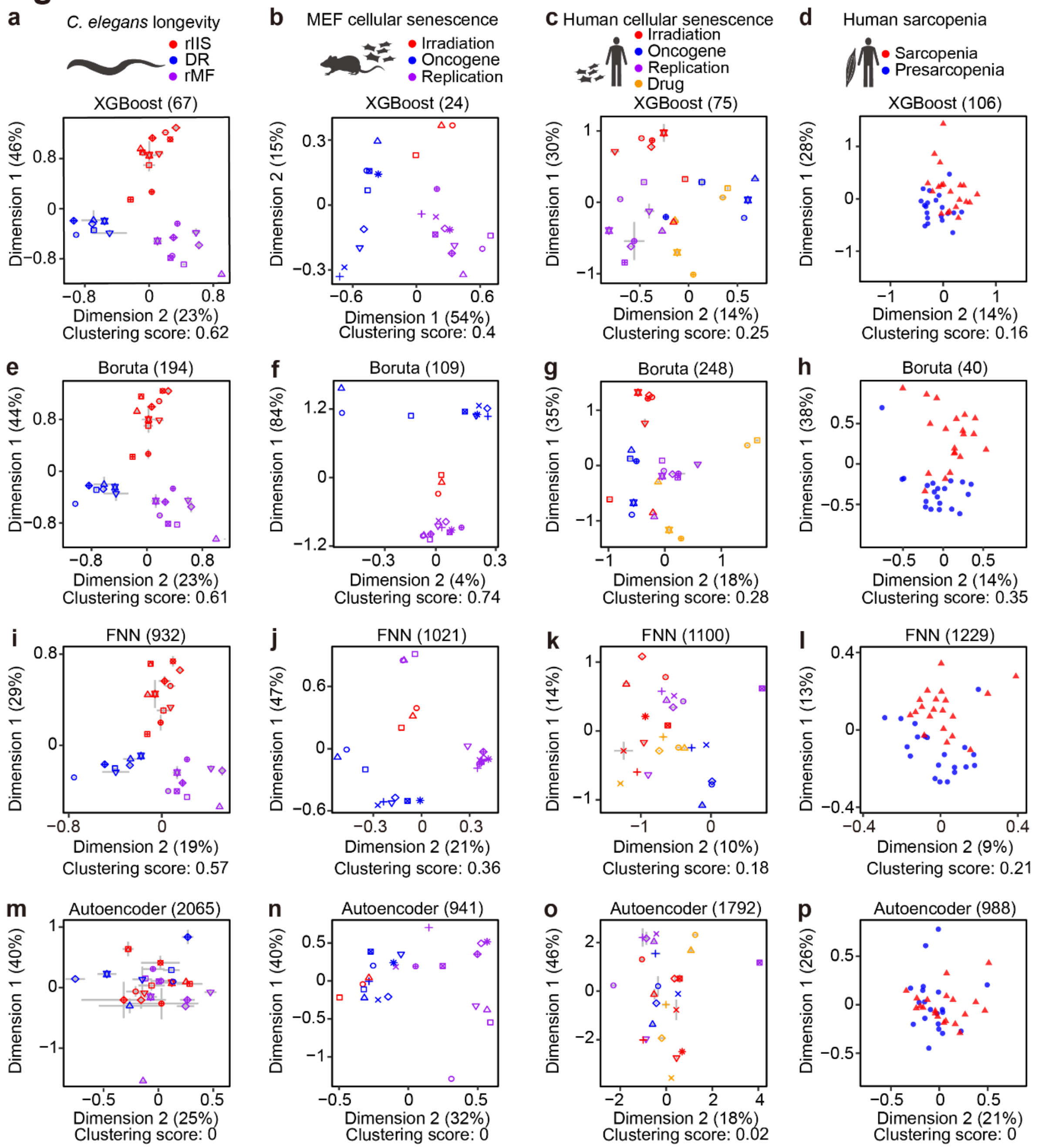

Figure S2. Transcriptome landscapes using four representative machine/deep learning-based tools in longevity-promoting regimens, cellular senescence, and sarcopenia. For machine learning-based approaches, XGBoost (Chen & Guestrin, 2016) and Boruta (Kursa, 2014) were used. For deep learning-based approaches, feedforward neural network (FNN, Candel et al., 2016) and autoencoder (Gulli & Pal, 2017) were used. (**a, e, i, m**) MDS plots of the transcriptomic changes caused by

three longevity-promoting regimens in *C. elegans*: reduced insulin/IGF-1 signaling pathway (rIIS, red), dietary restriction (DR, blue), and reduced mitochondrial function (rMF, purple) by using XGBoost (67 genes) (a), Boruta (194 genes) (e), FNN (932 genes) (i), and autoencoder (2065 genes) (m). (**b, f, j, n**) MDS plots of transcriptomic changes caused by cellular senescence inducers in mouse embryonic fibroblasts (MEF): irradiation (red), oncogene-induced senescence (blue), and extensive replication (purple) by using XGBoost (24 genes) (b), Boruta (109 genes) (f), FNN (1021 genes) (j), and autoencoder (941 genes) (n). (**c, g, k, o**) MDS plots of transcriptomic changes caused by cellular senescence inducers in human cells: irradiation (red), oncogene-induced senescence (blue), extensive replication (purple), and senescence-induced drug (orange) by using XGBoost (75 genes) (c), Boruta (248 genes) (g), FNN (1100 genes) (k), and autoencoder (1792 genes) (o). (**d, h, l, p**) MDS plots of transcriptomic changes caused by sarcopenia in human: sarcopenia (red) and presarcopenia (blue) by using XGBoost (106 genes) (d), Boruta (40 genes) (h), FNN (1229 genes) (l), and autoencoder (988 genes) (p).

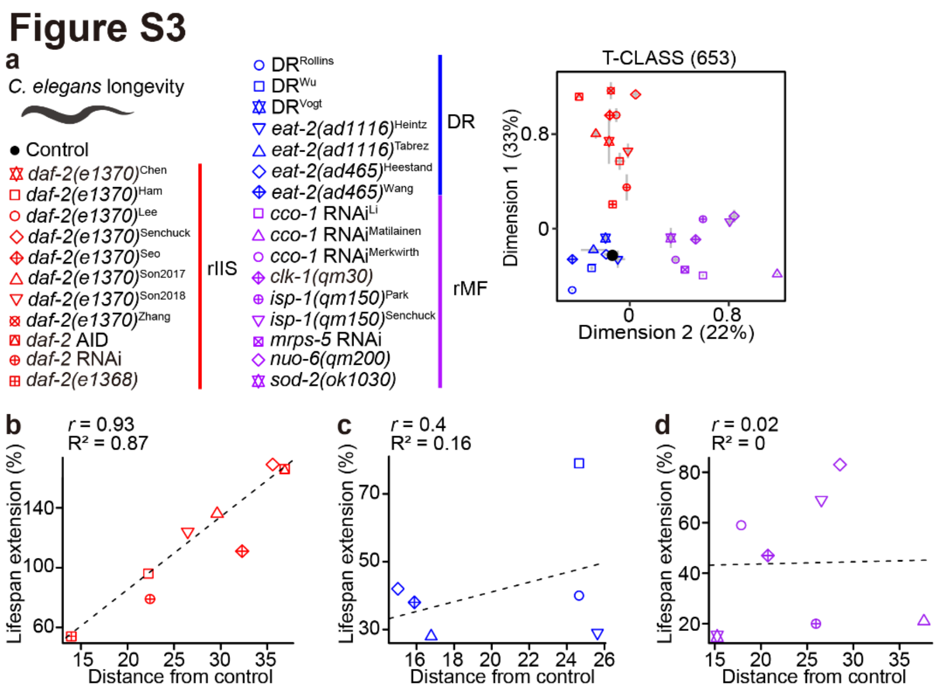

Figure S3. Correlation between extent of lifespan extension and Euclidean distance from the control in *C. elegans*. (**a**) An MDS plot of the three longevity-promoting regimens analyzed in *C. elegans*: rIIS (red), DR (blue), and rMF (purple) using T-CLASS including control. (**b**) The extent of lifespan extension by rIIS displayed positive correlation with Euclidean distance from the control. (**c, d**) Lifespan extension by DR (c) or rMF (d) did not show a significant positive correlation with the Euclidean distance from the control. The correlations were calculated by Pearson correlation analysis. *r*: correlation coefficient. R^2: coefficient of determination.

Figure S4

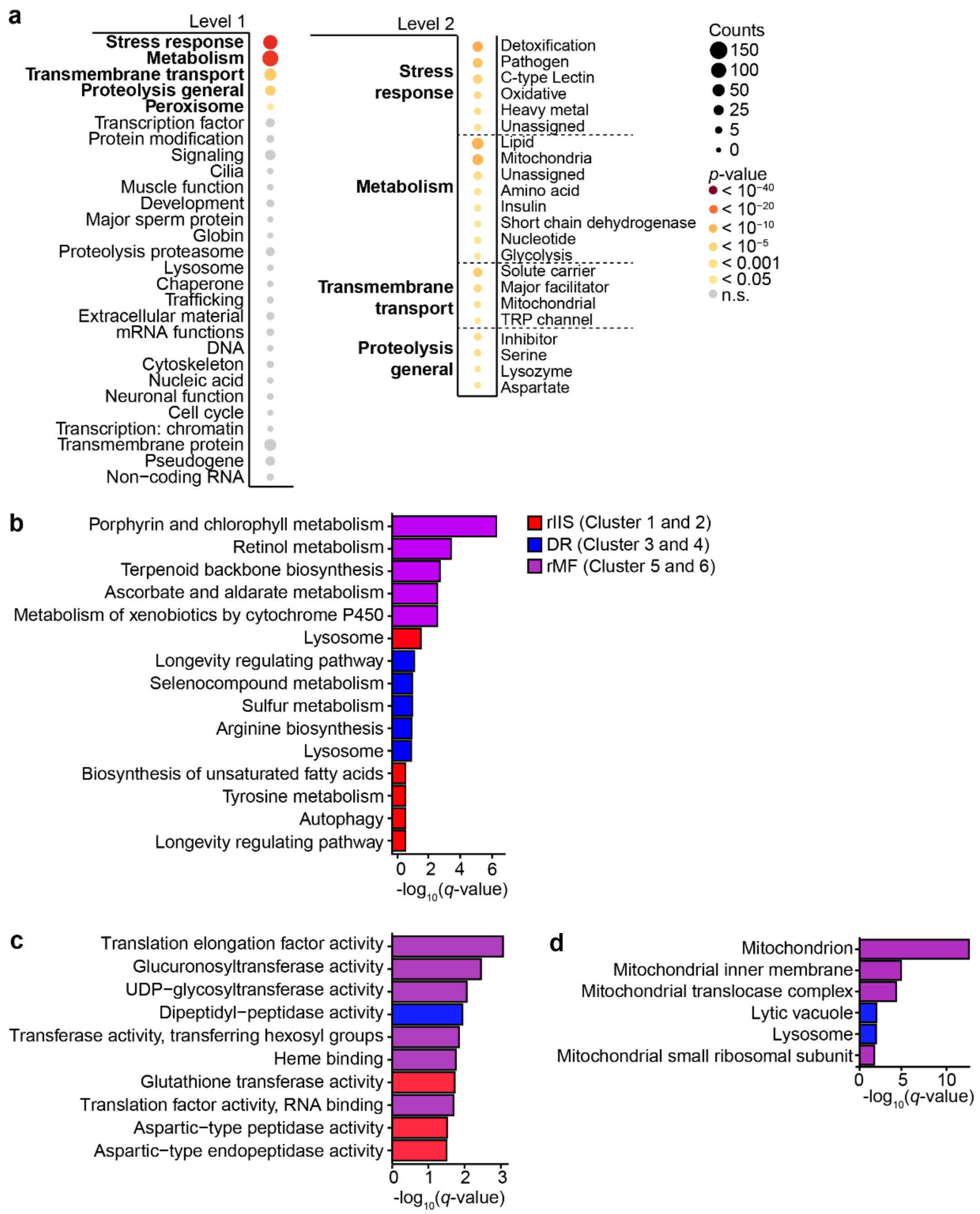

Figure S4. Gene set enrichment analysis of the optimal genes selected by using T-CLASS for *C. elegans* longevity. The analysis included WormCat (Holdorf et al., 2020), Kyoto Encyclopedia of Genes and Genomes (KEGG) pathway enrichment analysis (Kanehisa et al., 2017), and gene ontology (GO) term analysis for Cellular Component and Molecular Function (Yu et al., 2012). (**a**) WormCat terms enriched

among the optimal genes selected by T-CLASS. The term "Stress response" (Category level 1) includes "Detoxification" and "Pathogen", the established terms in longevity-promoting pathways (Cypser et al., 2006; Lee et al., 2021; Higgins et al., 2022; Holdorf et al., 2020). The term "Metabolism" (Category level 1) includes "insulin" and "mitochondria", which represent rIIS and rMF pathways, respectively. (**b**) Top five significantly enriched KEGG pathways ($q < 0.05$) for the three clusters of the optimal gene sets shown in Figure 3b. Each gene cluster is associated with biological functions represented by these enriched pathways (Calvert et al., 2016; Son et al., 2017; Kenyon, 2010; Ackerman & Gems, 2012; Yang and Hekimi, 2010; Oliveira et al., 2009). (**c**, **d**) GO term analysis for Cellular Component (c) and Molecular Function (d). The terms "Translation elongation" and "Mitochondria" are associated with rMF, "Lysosome" and "Dipeptidyl-peptidase activity" with DR, and "Glutathione transferase activity" with rIIS.

Figure S5

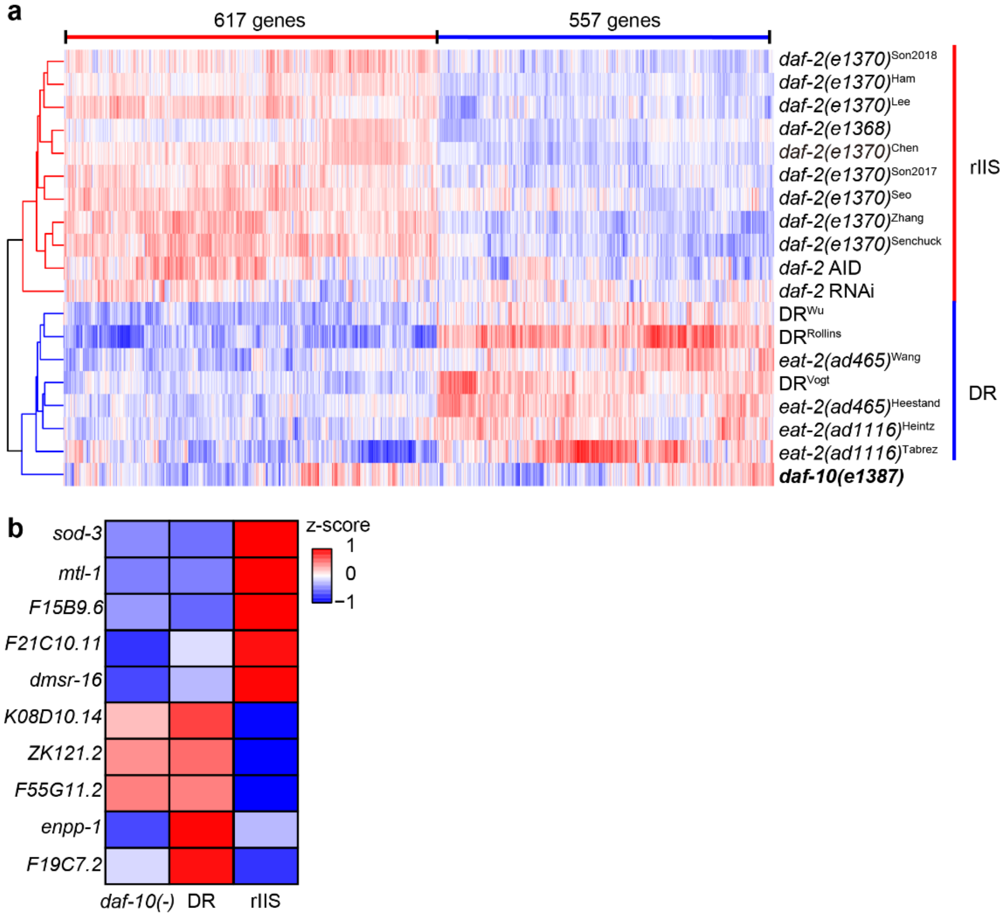

Figure S5. Dissecting the mixed effect of *daf-10(e1387)*. (**a**) Clustering heatmap of 1174 genes across long-lived mutants and conditions: rIIS, DR, and *daf-10(e1387)*. Count values were transformed using variance stabilizing transformation and converted to Z-scores. Hierarchical clustering was performed using Euclidean distance to group samples. (**b**) Expression patterns of top ten genes that most effectively distinguish DR and rIIS conditions (identified by T-CLASS based on the highest *F*-values), shown across DR, rIIS, and *daf-10(e1387)*. Eight out of the ten genes were similarly expressed ($p < 0.001$) in DR and *daf-10* mutants. Count values were transformed using variance stabilizing transformation and normalized into Z-scores. *p*-value was calculated using a log-rank (Mantel-Cox) test.

Figure S6

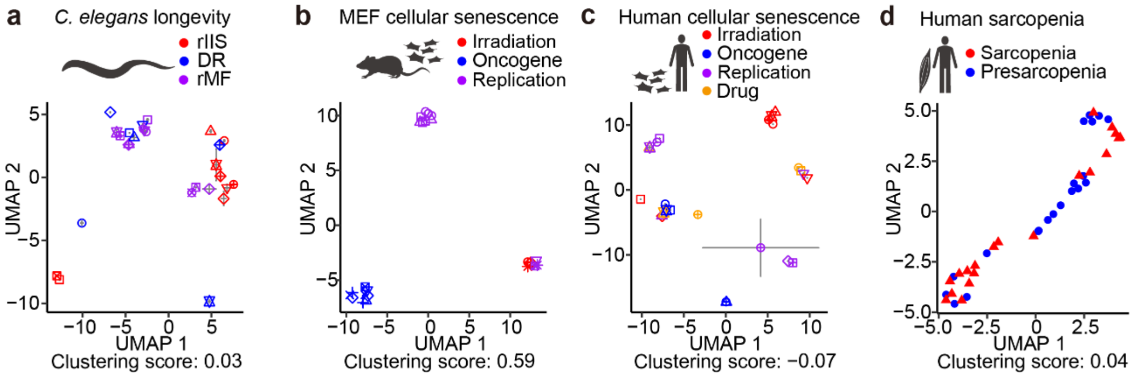

Figure S6. Transcriptome landscapes using Seurat across various species and biological contexts. The performance of categorization was evaluated by calculating the clustering score (silhouette coefficient). (**a**–**d**) UMAP (Uniform Manifold Approximation and Projection) plots showing the transcriptome clusters of the three longevity paradigms in *C. elegans* (a) (clustering score: 0.03), cellular senescence in MEF (b) (clustering score: 0.59) and human (c) (clustering score: −0.07), and human sarcopenia (d) (clustering score: 0.04). T-CLASS was superior or comparable to the result using Seurat based on clustering scores.

Supplementary references

1. Ackerman, D., & Gems, D. (2012). Insulin/IGF-1 and hypoxia signaling act in concert to regulate iron homeostasis in *Caenorhabditis elegans*. *PLoS Genet, 8*(3), e1002498. doi:10.1371/journal.pgen.1002498.
2. Calvert, S., Tacutu, R., Sharifi, S., Teixeira, R., Ghosh, P., & de Magalhaes, J. P. (2016). A network pharmacology approach reveals new candidate caloric restriction mimetics in *C. elegans*. *Aging Cell, 15*(2), 256-266. doi:10.1111/acel.12432.
3. Candel, A., V. Parmar, E. LeDell, and A. Arora. 2016. *Deep Learning With H_2O*, 1–21. H2O.Ai Inc.
4. Casella, G., Munk, R., Kim, K. M., Piao, Y., De, S., … Gorospe, M. (2019). Transcriptome signature of cellular senescence. *Nucleic Acids Res, 47*(14), 7294–7305. doi:10.1093/nar/gkz555.
5. Chen, A. T., Guo, C., Itani, O. A., Budaitis, B. G., Williams, T. W., Hopkins, C. E., . . . Hu, P. J. (2015). Longevity Genes Revealed by Integrative Analysis of Isoform-Specific *daf-16*/*FoxO* Mutants of *Caenorhabditis elegans*. *Genetics, 201*(2), 613-629. doi:10.1534/genetics.115.177998.
6. Chen, T., and C. Guestrin. 2016. *XGBoost: A Scalable Tree Boosting System*. Proceedings of the 22nd ACM SIGKDD International Conference on Knowledge Discovery and Data Mining.
7. Cheng, Y., Wang, S., Zhang, H., Lee, J. S., Ni, C., … Mendell, J. T. (2024). A non-canonical role for a small nucleolar RNA in ribosome biogenesis and senescence. *Cell, 187*(17), 4770–4789.e23. doi:10.1016/j.cell.2024.06.019.
8. Cypser, J. R., Tedesco, P., & Johnson, T. E. (2006). Hormesis and aging in *Caenorhabditis elegans*. Exp Gerontol, 41(10), 935-939.

https://doi.org/10.1016/j.exger.2006.09.004.

9. De Cecco, M., Criscione, S. W., Peckham, E. J., Hillenmeyer, S., Hamm, E. A., … Sedivy, J. M. (2019). L1 drives IFN-I in senescent cells and promotes age-related inflammation. *Nature*, *566*(7742), 73–78. doi:10.1038/s41586-018-0784-9.
10. Gallage, S., Irvine, E. E., Barragan Avila, J. E., Reen, V., Pedroni, S. M. A., Duran, I., . . . Withers, D. J. (2024). Ribosomal S6 kinase 1 regulates inflammaging via the senescence secretome. *Nat Aging, 4*(11), 1544-1561. doi:10.1038/s43587-024-00695-z.
11. Gulli, A., and S. Pal. 2017. *Deep Learning With Keras*. Packt Publishing Ltd.
12. Ham, S., Kim, S. S., Park, S., Kim, E. J. E., Kwon, S., Park, H. H., . . . Lee, S. V. (2022). Systematic transcriptome analysis associated with physiological and chronological aging in *Caenorhabditis elegans*. *Genome Res, 32*(11-12), 2003-2014. doi:10.1101/gr.276515.121.
13. Heestand, B. N., Shen, Y., Liu, W., Magner, D. B., Storm, N., Meharg, C., . . . Antebi, A. (2013). Dietary restriction induced longevity is mediated by nuclear receptor NHR-62 in *Caenorhabditis elegans*. *PLoS Genet, 9*(7), e1003651. doi:10.1371/journal.pgen.1003651.
14. Heintz, C., Doktor, T. K., Lanjuin, A., Escoubas, C., Zhang, Y., Weir, H. J., . . . Mair, W. B. (2017). Splicing factor 1 modulates dietary restriction and TORC1 pathway longevity in *C. elegans*. *Nature, 541*(7635), 102-106. doi:10.1038/nature20789.
15. Higgins, D. P., C. M. Weisman, D. S. Lui, F. A. D'Agostino, and A. K. Walker. 2022. Defining Characteristics and Conservation of Poorly Annotated Genes in *Caenorhabditis elegans* Using WormCat 2.0. *Genetics* 221,

no. 4: iyac085. https://doi.org/10.1093/genetics/iyac085.

16. Holdorf, A. D., D. P. Higgins, A. C. Hart, et al. 2020. WormCat: An Online Tool for Annotation and Visualization of *Caenorhabditis elegans* Genome-Scale Data. *Genetics* 214, no. 2: 279–294. https://doi.org/10.1534/genetics.119.302919.

17. Joung, J., Heo, Y., Kim, Y., Kim, J., Choi, H., … Kang, C. (2025). Cell enlargement modulated by GATA4 and YAP instructs the senescence-associated secretory phenotype. *Nat Commun, 16*, 1696. doi:10.1038/s41467-025-56929-0.

18. Kanehisa, M., M. Furumichi, M. Tanabe, Y. Sato, and K. Morishima. 2017. KEGG: New Perspectives on Genomes, Pathways, Diseases and Drugs. *Nucleic Acids Research* 45, no. D1: D353–D361. https://doi.org/10.1093/nar/gkw1092.

19. Kenyon, C. J. (2010). The genetics of ageing. *Nature, 464*(7288), 504-512. doi:10.1038/nature08980.

20. Kursa, M. B. 2014. Robustness of Random Forest-Based Gene Selection Methods. *BMC Bioinformatics* 15: 8. https://doi.org/10.1186/1471-2105-15-8.

21. Lee, Y., Jung, Y., Jeong, D. E., Hwang, W., Ham, S., Park, H. H., . . . Lee, S. V. (2021). Reduced insulin/IGF1 signaling prevents immune aging via ZIP-10/bZIP-mediated feedforward loop. *J Cell Biol, 220*(5). doi:10.1083/jcb.202006174.

22. Li, T. Y., Sleiman, M. B., Li, H., Gao, A. W., Mottis, A., Bachmann, A. M., . . . Auwerx, J. (2021). The transcriptional coactivator CBP/p300 is an evolutionarily conserved node that promotes longevity in response to mitochondrial stress. *Nat Aging, 1*(2), 165-178. doi:10.1038/s43587-020-00025-z.

23. Marthandan, S., Baumgart, M., Priebe, S., Groth, M., Schaer, J., … Hemmerich, P. (2016). Conserved senescence associated genes and pathways in primary human fibroblasts detected by RNA-Seq. *PLoS ONE, 11*(5), e0154531.

doi:10.1371/journal.pone.0154531.

24. Matilainen, O., Sleiman, M. S. B., Quiros, P. M., Garcia, S., & Auwerx, J. (2017). The chromatin remodeling factor ISW-1 integrates organismal responses against nuclear and mitochondrial stress. *Nat Commun, 8*(1), 1818.

25. Merkwirth, C., Jovaisaite, V., Durieux, J., Matilainen, O., Jordan, S. D., Quiros, P. M., . . . Dillin, A. (2016). Two Conserved Histone Demethylases Regulate Mitochondrial Stress-Induced Longevity. *Cell, 165*(5), 1209-1223. doi:10.1016/j.cell.2016.04.012.

26. Oliveira, R. P., Porter Abate, J., Dilks, K., Landis, J., Ashraf, J., Murphy, C. T., & Blackwell, T. K. (2009). Condition-adapted stress and longevity gene regulation by *Caenorhabditis elegans* SKN-1/Nrf. *Aging Cell, 8*(5), 524-541. doi:10.1111/j.1474-9726.2009.00501.x.

27. Papaspyropoulos, A., Hazapis, O., Altulea, A., Polyzou, A., Verginis, P., … Gorgoulis, V. (2023). Decoding of translation-regulating entities reveals heterogeneous translation deficiency patterns in cellular senescence. *Aging Cell, 22*(9), e13893. doi:10.1111/acel.13893.

28. Petriv, O. I., Tang, L., Titorenko, V. I., & Rachubinski, R. A. (2004). A New Definition for the Consensus Sequence of the Peroxisome Targeting Signal Type 2. *Journal of Molecular Biology, 341*(1), 119-134. doi:https://doi.org/10.1016/j.jmb.2004.05.064

29. Purcell, J. W., A. Kruger, and M. A. Tainsky. 2014. Gene Expression Profiling of Replicative and Induced Senescence. *Cell Cycle* 13, no. 24: 3927–3937. https://doi.org/10.4161/15384101.2014.973327.

30. Roitenberg, N., Bejerano-Sagie, M., Boocholez, H., Moll, L., Marques, F. C., Golodetzki, L., . . . Cohen, E. (2018). Modulation of caveolae by insulin/IGF-1

signaling regulates aging of *Caenorhabditis elegans*. *EMBO Rep, 19*(8). doi:10.15252/embr.201745673.

31. Rollins, J. A., Shaffer, D., Snow, S. S., Kapahi, P., & Rogers, A. N. (2019). Dietary restriction induces posttranscriptional regulation of longevity genes. *Life Sci Alliance, 2*(4). doi:10.26508/lsa.201800281.
32. Savić, R., Yang, J., Koplev, S., An, M. C., Patel, P. L., … Argmann, C. (2023). Integration of transcriptomes of senescent cell models with multi-tissue patient samples reveals reduced COL6A3 as an inducer of senescence. *Cell Reports, 42*(11), 113371. doi:10.1016/j.celrep.2023.113371.
33. Senchuk, M. M., Dues, D. J., Schaar, C. E., Johnson, B. K., Madaj, Z. B., Bowman, M. J., . . . Van Raamsdonk, J. M. (2018). Activation of DAF-16/FOXO by reactive oxygen species contributes to longevity in long-lived mitochondrial mutants in *Caenorhabditis elegans*. *PLoS Genet, 14*(3), e1007268. doi:10.1371/journal.pgen.1007268.
34. Seo, M., Seo, K., Hwang, W., Koo, H. J., Hahm, J. H., Yang, J. S., . . . Lee, S. J. (2015). RNA helicase HEL-1 promotes longevity by specifically activating DAF-16/FOXO transcription factor signaling in *Caenorhabditis elegans*. *Proc Natl Acad Sci U S A, 112*(31), E4246-4255. doi:10.1073/pnas.1505451112.
35. Son, H. G., Seo, K., Seo, M., Park, S., Ham, S., An, S. W. A., . . . Lee, S. V. (2018). Prefoldin 6 mediates longevity response from heat shock factor 1 to FOXO in *C. elegans*. *Genes Dev, 32*(23-24), 1562-1575. doi:10.1101/gad.317362.118.
36. Son, H. G., Seo, M., Ham, S., Hwang, W., Lee, D., An, S. W., . . . Lee, S. V. (2017). RNA surveillance via nonsense-mediated mRNA decay is crucial for longevity in daf-2/insulin/IGF-1 mutant *C. elegans*. *Nat Commun, 8*, 14749.

doi:10.1038/ncomms14749.
37. Sturmlechner, I., Sine, C. C., Jeganathan, K. B., Zhang, C., Fierro Velasco, R. O., Baker, D. J., . . . van Deursen, J. M. (2022). Senescent cells limit p53 activity via multiple mechanisms to remain viable. *Nat Commun, 13*(1), 3722. doi:10.1038/s41467-022-31239-x.
38. Tabrez, S. S., Sharma, R. D., Jain, V., Siddiqui, A. A., & Mukhopadhyay, A. (2017). Differential alternative splicing coupled to nonsense-mediated decay of mRNA ensures dietary restriction-induced longevity. *Nat Commun, 8*(1), 306. doi:10.1038/s41467-017-00370-5.
39. Vogt, M. C., & Hobert, O. (2023). Starvation-induced changes in somatic insulin/IGF-1R signaling drive metabolic programming across generations. *Science Advances, 9*(14), eade1817. doi:doi:10.1126/sciadv.ade1817.
40. Wang, Z., Zou, L., Zhang, Y., Zhu, M., Zhang, S., Wu, D., . . . Chen, D. (2023). ACS-20/FATP4 mediates the anti-ageing effect of dietary restriction in *C. elegans*. *Nat Commun, 14*(1), 7683. doi:10.1038/s41467-023-43613-4.
41. Yang, W., & Hekimi, S. (2010). A mitochondrial superoxide signal triggers increased longevity in *Caenorhabditis elegans*. *PLoS Biol, 8*(12), e1000556. doi:10.1371/journal.pbio.1000556.
42. Yang, L., You, J., Yang, X., Jiao, R., Xu, J., … Liu, P. (2025). ACSS2 drives senescence-associated secretory phenotype by limiting purine biosynthesis through PAICS acetylation. *Nat Commun, 16*(1), 2071. doi:10.1038/s41467-025-57334-3.
43. Yu, G., L.-G. Wang, Y. Han, and Q.-Y. He. 2012. clusterProfiler: An R Package for Comparing Biological Themes Among Gene Clusters. *OMICS: A Journal of Integrative Biology* 16, no. 5: 284–287. https://doi.org/10.1089/omi.2011.0118

44. Zhang, Y. P., Zhang, W. H., Zhang, P., Li, Q., Sun, Y., Wang, J. W., . . . Dong, M. Q. (2022). Intestine-specific removal of DAF-2 nearly doubles lifespan in *Caenorhabditis elegans* with little fitness cost. *Nat Commun, 13*(1), 6339. doi:10.1038/s41467-022-33850-4.

45. Zuo, X., Zhao, R., Wu, M., Yin, J., Pan, L., Chen, Y., . . .Sun, L. (2025). Multi-omic profiling of sarcopenia identifies disrupted branched-chain amino acid catabolism as a causal mechanism and therapeutic target. *Nature Aging*, *5*, 419–436. doi:10.1038/s43587-024-00797-8.

Supplementary Tables

Table S1. Information of public RNA-seq datasets

Table S2. Gene sets obtained from ten XGBoost runs

Table S3. Information of top 653 genes

Table S4. Gene information and Z-score of six clusters

Table S5. Spearman's correlation between query data and longevity-promoting regimens

Table S6. Lifespan data
