# Supplementary material for: T‐CLASS: An Online Tool for the Identification and Classification of Aging and Senescence Using Transcriptome Data
Source: Aging Cell. 2025 Aug 14;24(10):e70193. doi: 10.1111/acel.70193 (PMC12507419; doi:10.1111/acel.70193)
Supplement: Supplementary file 2 — Appendix S2: acel70193‐sup‐0002‐AppendixS2.pdf. [file ACEL-24-e70193-s007.pdf]

Manual for T-CLASS

T-CLASS: Transcriptomic CLassification via Adaptive learning of Signature States

Please cite this paper as Lee et al., *Aging Cell*, 2025 (doi:10.1111/acel.70193) when using T-CLASS.

1. Accessing T-CLASS

- Visit the T-CLASS website (http://www.t-class.kaist.ac.kr).

2. Register and login to T-CLASS

- To upload a query and/or reference count matrix to T-CLASS, login is required.

***Registration is required in accordance with KAIST institutional server security policies. The process is simple and requires minimal effort, without the need to submit any personal information.

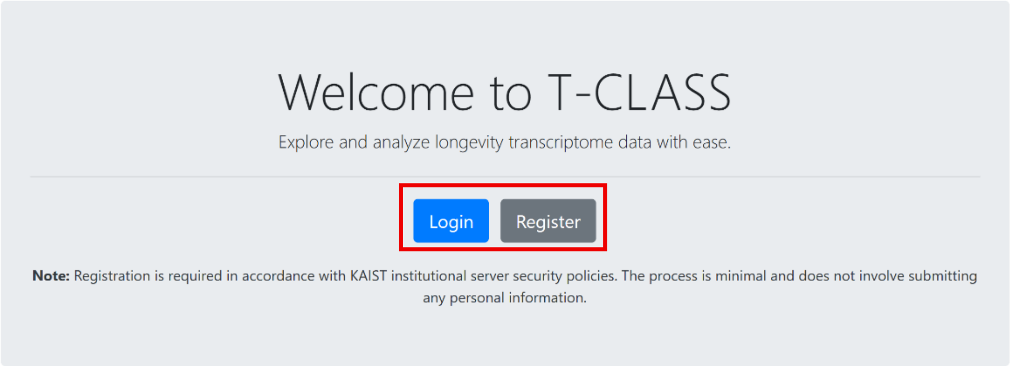

2.1. Register

: Create an account before logging in to T-CLASS.

2.2. Login

: After registering an account, login to access Upload and analysis features.

3. Upload and Analyze Transcriptome

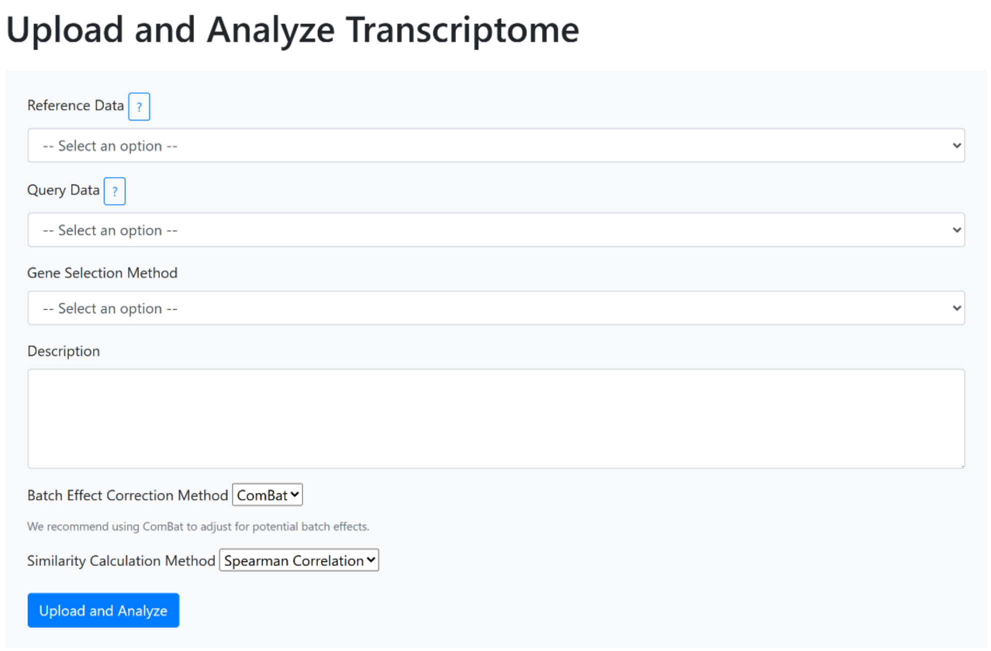

- After logging in, users are redirected to the Upload and Analyze Transcriptome page.
- Users can upload a query and reference (optional) count matrix. Example input files ("Sample reference file.txt" and "Sample query file.txt") can be found in the Introduction page.

3.1. Reference Data

: Users can either upload their manually created reference data or select pre-uploaded reference data for *C. elegans* longevity and human senescence.

3.2. Query Data

***Please follow the format below when uploading the query data. Use exactly three underscores as separators for column names.

1) A tab-delimited .txt format with raw count values.

2) Ensembl Gene ID provided as row names.

3) Column names must follow the format below.

3-1) First column name: “target_id”

3-2) Control group column names: “[UserName]_control_control_[number]”

e.g.) Lee_control_control_1, Lee_control_control_2, Lee_control_control_3

3-3) Experimental group column names: “[UserName]_query_query_[number]”

e.g.) Lee_query_query_1, Lee_query_query_2, Lee_query_query_3

→ An example of a query data should look like this.

target_id	Lee_control_control_1	Lee_control_control_2	Lee_control_control_3	Lee_query_query_1	Lee_query_query_2	Lee_query_query_3
WBGene00000001	67	37	28	5	7	3
WBGene00000002	7	0	0	0	3	4
WBGene00000003	111	88	118	197	178	163
WBGene00000004	25	13	14	4	4	5
WBGene00000005	42	38	46	89	116	108
WBGene00000006	15	24	22	81	66	55
WBGene00000007	2	0	3	5	3	1
WBGene00000008	15	12	28	28	36	40
WBGene00000009	23	15	17	28	28	37
WBGene00000010	258	173	211	43	33	42
WBGene00000012	1	0	0	5	7	3
WBGene00000013	12	9	18	219	212	297
WBGene00000014	0	0	0	0	0	0
WBGene00000015	0	3	0	0	2	0
WBGene00000016	37	22	41	99	80	76
WBGene00000017	43	30	37	115	83	135
WBGene00000018	24	39	37	42	18	27
WBGene00000019	3	0	2	0	0	0
WBGene00000020	2	2	2	6	1	1
...	...	...	...	...	...	...

3.3. Gene Selection Method

: Users can select either T-CLASS or Boruta to classify query data with the most suitable option for their dataset.

3.4. Batch Effect Correction Method

: Users can choose to apply batch correction using ComBat or proceed without correction by selecting None. By default, we recommend using ComBat to adjust for potential batch effects.

3.5. Similarity Calculation Method

: Users can calculate similarity among samples by using Spearman Correlation.

4. Analysis in Progress

◆ If data are uploaded properly, the analysis will proceed. The progress of the analysis will be shown as below.

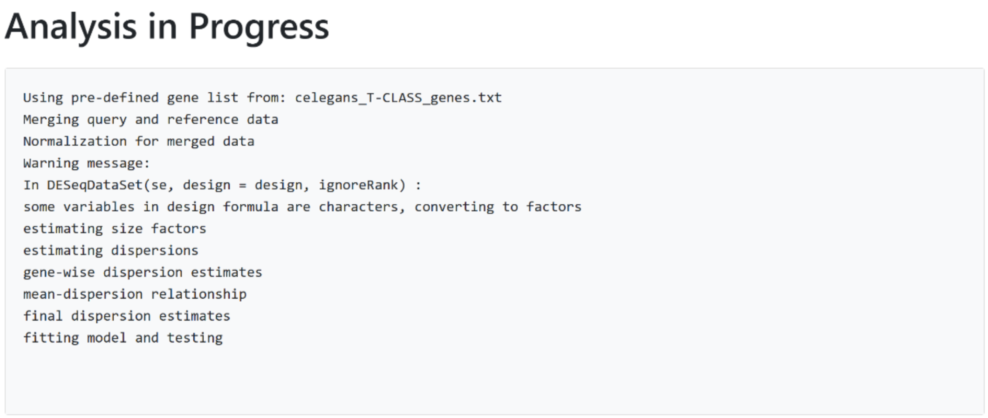

5. Analysis Result

◆ When the analysis is complete, users will be redirected to the Analysis Result page.

5.1. Transcriptome Details

: This section provides a summary of the input parameters and analysis settings (Gene Selection method, Batch Correction method, and Distance Metric) for the submitted job.

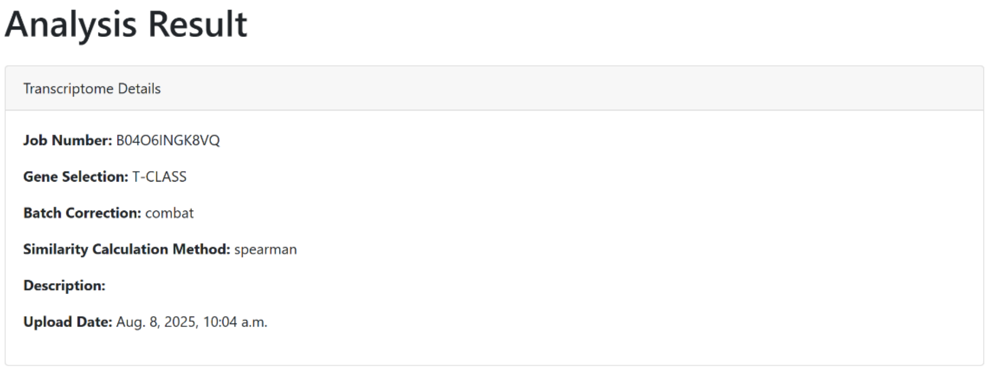

5.2. Transcriptomic landscape and quantitative comparison

Transcriptomic landscape & quantitative comparison

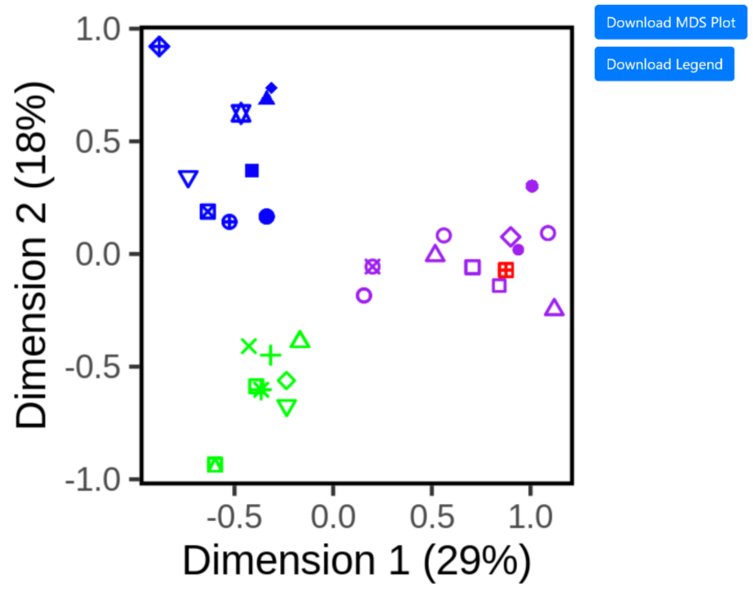

Pathway	Similarity
rMF	0.548
**rIIS**	**0.640**
DR	0.589

: This section displays a Multidimensional scaling (MDS) plot that visualizes the transcriptomic similarity between query and reference samples in a two-dimensional space.

: The computed similarity scores by using Spearman correlation, which are used to classify the query samples among the predefined reference categories, are presented in a table.

: Users can download the MDS plot and the corresponding figure legend in PDF format by clicking “Download MDS Plot” and “Download Legend”, respectively. The downloaded PDF files can be modified by users for further presentation or publication purposes.

5.3. Heatmap of Gene Expression

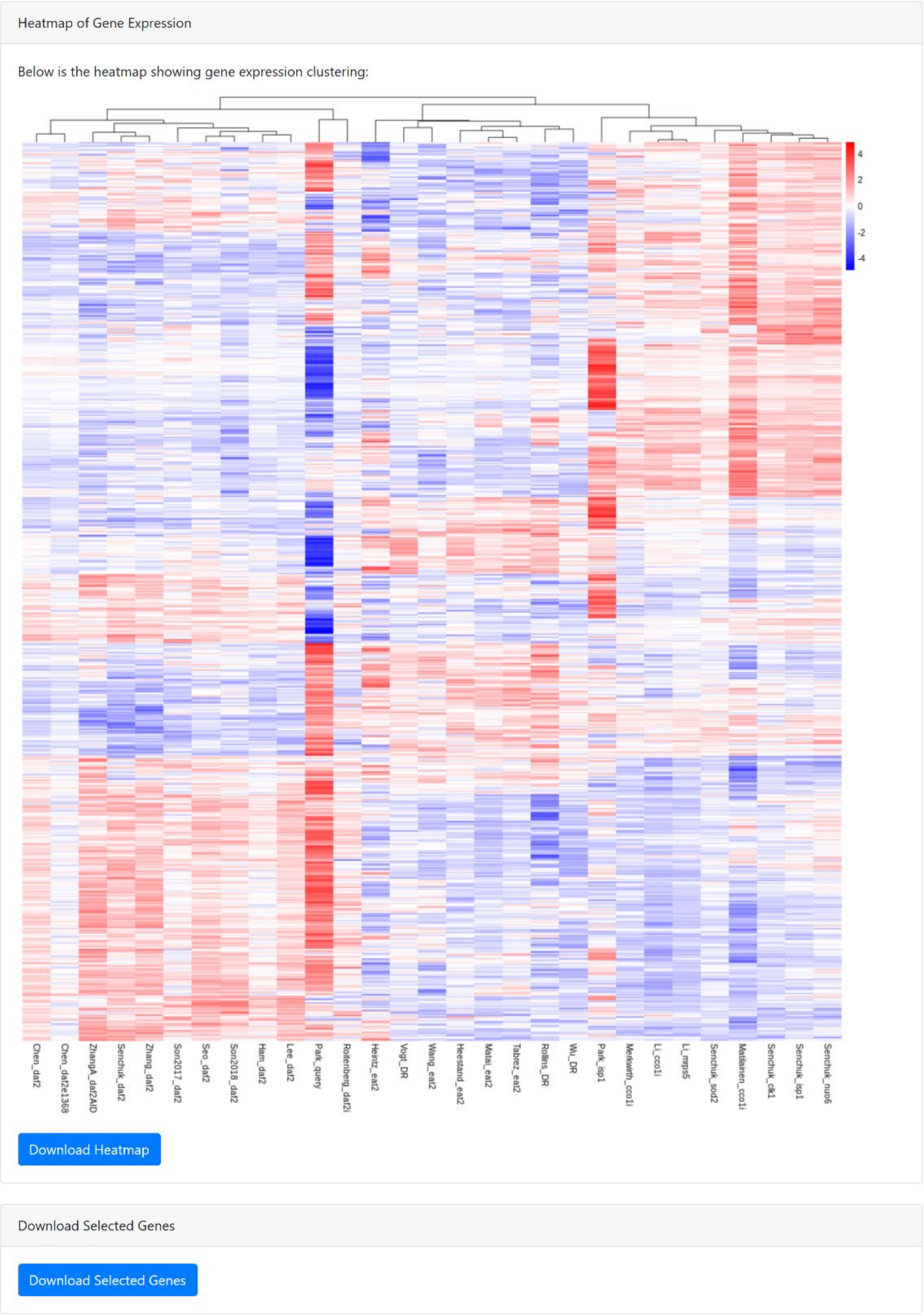

: T-CLASS provides a result of heatmap analysis that visualizes the overall gene expression, based on the optimal gene set. Heatmap can be downloaded in PDF format by clicking “Download Heatmap”.

5.4. Download Selected Genes

: The optimal gene set used for the analysis can be downloaded in TXT format by the user by clicking “Download Selected Genes”.
