# Supplementary material for: T‐CLASS: An Online Tool for the Identification and Classification of Aging and Senescence Using Transcriptome Data
Source: Aging Cell. 2025 Aug 14;24(10):e70193. doi: 10.1111/acel.70193 (PMC12507419; doi:10.1111/acel.70193)
Supplement: Supplementary file 8 — Table S6: acel70193‐sup‐0008‐TableS6.docx. [file ACEL-24-e70193-s002.docx]

**Table S6.** **Lifespan data**

| **Strain/treatment** | **Mean lifespan** ± **s.e.m. (days)** | **75th percentile** | **% lifespan change** | **Number of animals that died/total** | ***p* value vs. control** | **Figure in text** |
| --- | --- | --- | --- | --- | --- | --- |
| N2/fed control | 14.3±0.38 | 17 |  | 116/125 |  | Fig. 6c |
| N2/fed rifampicin | 22.12±0.64 | 26 | 54.7% | 77/120 | <0.001 | Fig. 6c |
| N2/fed adult-only rifampicin | 24.06±0.55 | 29 | 68.3%  (8.8%^fed rifampicin^) | 66/120 | <0.001  (0.09^fed rifampicin^) | Fig. 6c |
| N2/fed larva-only rifampicin | 16.78±0.39 | 20 | 17.3% | 101/125 | <0.001 | Fig. 6c |
| N2/fed control | 17.6±0.33 | 21 |  | 116/125 |  |  |
| N2/fed rifampicin | 29.41±0.77 | 35 | 67.1% | 80/100 | <0.001 |  |
| N2/fed adult-only rifampicin | 28.27±0.61 | 31 | 60.6%  (-3.9%^fed rifampicin^) | 45/75 | <0.001  (0.06^fed rifampicin^) |  |
| N2/fed larva-only rifampicin | 19.34±0.32 | 21 | 9.9% | 111/125 | <0.001 |  |
| N2/FD | 21.93±0.7 | 26 |  | 92/180 |  | Fig. 6d |
| N2/FD rifampicin | 22.31±0.75 | 26 | 1.7% | 103/150 | 0.5 | Fig. 6d |
| N2/FD | 31.96±0.61 | 38 |  | 127/180 |  |  |
| N2/FD rifampicin | 33.57±0.69 | 38 | 5.0% | 118/150 | <0.05 |  |
| N2/fed control | 20.05±0.38 | 23 |  | 111/120 |  | Fig. 6e |
| N2/fed atracurium | 23.47±0.39 | 26 | 17.1% | 128/150 | <0.001 | Fig. 6e |
| N2/fed adult-only atracurium | 22.77±0.35 | 26 | 13.6%  (-3.0%^fed atracurium^) | 130/150 | <0.001  (<0.05^fed atracurium^) | Fig. 6e |
| N2/fed larva-only atracurium | 19.97±0.31 | 23 | -0.4%  (-14.9%^fed atracurium^) | 143/150 | 0.6 (<0.001^fed atracurium^) | Fig. 6e |
| N2/fed control | 20.01±0.59 | 27 |  | 139/150 |  |  |
| N2/fed atracurium | 23.63±0.71 | 31 | 18.1% | 106/150 | <0.001 |  |
| N2/fed adult-only atracurium | 23.71±0.64 | 31 | 0.3%  (-3.0%^fed atracurium^) | 127/150 | <0.001  (0.6^fed atracurium^) |  |
| N2/fed larva-only atracurium | 21.81±0.6 | 27 | 9.0%  (-7.7%^fed atracurium^) | 130/150 | <0.05  (<0.05^fed atracurium^) |  |
| N2/FD | 22.82±0.75 | 26 |  | 49/123 |  | Fig. 6f |
| N2/FD atracurium | 18.54±0.62 | 21 | -18.8% | 39/125 | <0.001 | Fig. 6f |
| N2/FD | 19.93±0.79 | 27 |  | 56/125 |  |  |
| N2/FD atracurium | 15.37±0.32 | 17 | -22.9% | 36/125 | <0.001 |  |

Different experimental sets are distinguished by double-solid lines,and biological replicates within the same experimental set are distinguished by single-solid lines. Biological replicate experiments were performed in parallel, and statistical analysis was performed within each replicate experiment. Percent lifespan changes and *p* values were calculated against control conditions. *p* values were calculated using Mantel-Cox log-rank test.

^fed rifampicin/atracurium^: percent lifespan change or *p* value against rifampicin/atracurium-treated fed animals within the same experimental set.
